# Role of external forces in the mechanobiology of stem and differentiated chondrogenic cells embedded in a tissue-engineered construct for cartilage repair

**DOI:** 10.1186/s12929-026-01247-w

**Published:** 2026-05-04

**Authors:** Maria Carolina Cordeiro, Andrea Barbero, Ivan Martin, Martin J. Stoddart

**Affiliations:** 1 AO Research Institute, Davos, Switzerland; 2 Faculty of Medicine, University of Basel, Basel, Switzerland; 3 Department of Biomedicine, University of Basel, University Hospital Basel, Basel, Switzerland

**Keywords:** Articular cartilage, Mechanobiology, Regenerative rehabilitation, Tissue engineering, Mechanical stimuli, Post-traumatic osteoarthritis

## Abstract

Articular cartilage, as a mechanosensitive tissue, supports and distributes various mechanical forces—including compression, shear, hydrostatic pressure, and tensile strain—during joint loading and motion. These external forces deform not only the chondrocytes but also their pericellular matrix and the surrounding extracellular matrix (ECM). Those mechanical cues are detected by mechanosensors on the plasma membrane (e.g., integrins) and transmitted through the cytoskeleton, ultimately being converted into biochemical signals. These signals activate key mechanoresponsive intracellular pathways—including TGF-β-induced SMAD, Rho-GTPase, MAPKs (ERK, JNK, p38), PI3K/AKT/mTOR, MRTF-SRF, and YAP/TAZ—that regulate chondrogenic differentiation and cartilage-specific matrix synthesis. This field of study is known as mechanobiology. Over the past decades, it has gained increasing recognition, particularly with the emergence of tissue-engineering constructs as a novel strategy for cartilage repair. However, progress in chondrogenic mechanobiology has primarily centred on intrinsic substrate- or matrix-derived cues, while overlooking the role of extrinsic mechanical forces. This review therefore provides an updated perspective on chondrogenic mechanobiology, with a particular focus on the cellular responses to external mechanical stimuli. It also emphasizes the therapeutic potential of incorporating mechanical stimulation into tissue-engineering strategies for cartilage repair, an emerging filed referred to as Regenerative Rehabilitation (RR). Since this concept has so far been investigated mainly in vitro, we highlight only those studies and refer to it as In vitro Regenerative Rehabilitation. Moreover, this review also addresses post-traumatic osteoarthritis (PTOA), a common joint disorder that frequently results from traumatic cartilage damage. It explores the mechanobiological mechanisms underlying OA and discusses in vitro regenerative rehabilitation studies, highlighting how external forces could serve as an alternative to conventional biochemical treatments for preventing OA progression.

## Introduction

Cell function is tightly regulated by the surrounding microenvironment, which provides biochemical signals (e.g., hormones, growth factors, cytokines) and biophysical cues from the extracellular matrix (ECM), including topography, stiffness, and porosity. In addition, the local microenvironment transmits and is affected by mechanical forces like compression and shear stress. Over the past few decades, many studies have focused on exploring biochemical cues while overlooking biophysical factors. The study of biophysical factors has emerged as a significant field known as mechanobiology. Cells sense mechanical cues through a group of membrane-anchored receptors, such as integrins [41, 314], cadherins[57], cell-membrane-spanning G-protein-coupled receptors (GPCRs) [136, 222], primary cilia [115, 348] and mechanically activated ion channels [81, 123, 124]. In response, cells induce changes in protein conformation or enzyme-catalysed reactions or channel opening, which are then translated into biochemical signals, initiating downstream mechanosensing pathways. This process, known as mechanotransduction, plays a critical role in regulating cellular functions such as proliferation, migration, differentiation, and apoptosis. Through its feedback mechanisms, it ensures the maintenance of tissue homeostasis by changing the initial mechanical input [300]. Dysregulation of the mechanotransduction process can disrupt cellular homeostasis and contribute to the progression of pathological states, such as the case of Osteoarthritis (OA) [119]. Given the limited research on these biophysical factors, it is essential to develop a comprehensive understanding of how mechanical cues are sensed by cells, how these cues lead to the activation of specific molecular signalling pathways, and how these pathways ultimately regulate cellular functions, influence cell fate, and contribute to tissue homeostasis.

Mechanosensitive tissues, such as articular cartilage (AC), receive and dissipate external mechanical forces generated by movement and weight-bearing activities. AC, a 2—5 mm thick tissue, covers the ends of long bones, providing low-friction properties for joint articulation. It lacks blood vessels, nerves, and a lymphatic system, and is characterized by its robust biomechanical properties. These properties are attributed to the structure, composition and organization of AC, which is composed of chondrocytes embedded in ECM. The main components of ECM are a collagen network (type II, IX and XI), proteoglycans and water. The presence of negatively charged proteoglycans generates osmotic swelling pressure, which attracts water into the AC. Chondrocytes are the mainly responsible for the production and maintenance of the ECM. These cells are also subjected to multiaxial external forces involving compression, shear stress, hydrostatic pressure and tensile strain [100]. They are also surrounded by a specialized matrix region known as the pericellular matrix (PCM), which is distinct from the ECM due to the presence of collagen type VI and functions as a mediator of extracellular matrix– or microenvironment–derived mechanotransduction [340].

AC has limited self- repair capacity, which is largely attributed to its above-mentioned inherent characteristics. Current treatments in clinical practice for chondral lesions include autograft and allograft transplantation [296], microfracture [281] and autologous chondrocyte implantation [247]. Although these treatments may provide initial symptomatic relief, they often fail to produce lasting outcomes, typically resulting in the formation of fibrocartilaginous tissue with inferior biomechanical properties. Consequently, current research increasingly focuses on tissue engineering (TE) strategies aimed at recreating native AC by combining the tuning of biomaterial features—particularly their form (e.g., hydrogel or sponge), type (e.g., natural, synthetic, or hybrid), and biomechanical properties (e.g., stiffness, pore size, viscoelasticity, cell-adhesion ligands, and topography)—with chondrogenic cells such as articular chondrocytes (ACh) or nasal chondrocytes (NChs) or mesenchymal stromal cells (MSCs) or articular cartilage-derived progenitor cells (ACPCs). Following cartilage repair, patients typically undergo postoperative rehabilitation, which may involve either traditional full joint immobilization (rest) or passive joint movement provided by a continuous passive motion (CPM) device. Studies have demonstrated that full joint immobilization negatively affects the biochemical properties of cartilage, such as reducing chondrocyte biosynthetic activity and proteoglycan content [104]. Studies using rabbit models have shown that CPM after AC injury leads to increased hyaline cartilage content and reduced fibrous tissue formation [151]. This emphasizes the need to integrate motion as a fundamental element in cartilage repair strategies. In light of this, the field of Regenerative Rehabilitation (RR) has emerged, combining regenerative medicine techniques (e.g., tissue-engineered constructs) with fine-tuned rehabilitation protocols to enhance cartilage repair [10].

Over the past few decades, extensive in vitro and in vivo research has demonstrated that both chondrogenic and mechanically induced chondrogenic responses are mediated by focal adhesion complexes [252, 258] and are highly dependent on cytoskeletal organization ([1, 24, 345, 346]). Multiple molecular pathways have been implicated in these processes, including the SMAD-dependent pathway [30, 111] activated by transforming growth factor-beta (TGF-β), as well as several SMAD-independent pathways involving Rho-GTPase [324, 349], MAPKs (ERK, JNK, and p38) [172, 179, 180, 195, 239, 331], PI3K/AKT/mTOR [150, 299], MRTF-SRF [188], and YAP/TAZ signalling [63, 171, 176, 353, 354]. In parallel, substantial advances have been made in the field of RR, particularly through in vitro studies utilizing customized bioreactors that mimic joint biomechanics by applying isolated or combined mechanical forces. These studies have also explored a wide range of biomaterials and cell sources. Overall, these studies have demonstrated the potential of mechanical stimulation in promoting chondrogenesis and enhancing the expression of cartilage-like matrix proteins. As for in vivo research, to the best of our knowledge, only one study to date has reported findings within the context of RR. In this study, Chang et al. demonstrated that rabbits implanted with poly-lactic-co-glycolic acids (PLGA) scaffolds and treated with CPM developed cartilage with a hyaline-like phenotype and exhibited abundant glycosaminoglycan (GAG) production. In contrast, animals that underwent immobilization following implantation formed fibrocartilaginous tissue with modest GAG content [45]. Although external mechanical stimuli may serve as a valuable component in the treatment of chondral defects, they are also essential for maintaining healthy cartilage function, and abnormal mechanical loading or traumatic injury can contribute to the development of OA or post-traumatic OA. As no cure for OA currently exists (only symptomatic treatments) research has focused on fundamental questions, revealing that OA pathology is associated with alterations in mechanosensory proteins [25, 315] and dysregulation of molecular signalling pathways. In addition to the widely explored biochemical interventions, emerging therapeutic strategies may include RR or purely mechanical-based therapies. In vitro findings support this approach, demonstrating that mechanical stimulation can activate anti-inflammatory and anabolic responses.

This review is divided into two main sections: the first addresses fundamental questions concerning the role of subcellular mechanosensory components in chondrogenesis and the influence of external mechanical forces, along with insights into relevant molecular pathways. The second section focuses on in vitro research into Regenerative Rehabilitation therapies and their potential to promote cartilage formation, alongside the current insights into the mechanobiology of OA and physiotherapy approaches that may help in its prevention and treatment.

## Articular cartilage composition and structure

**Fig. 1:**
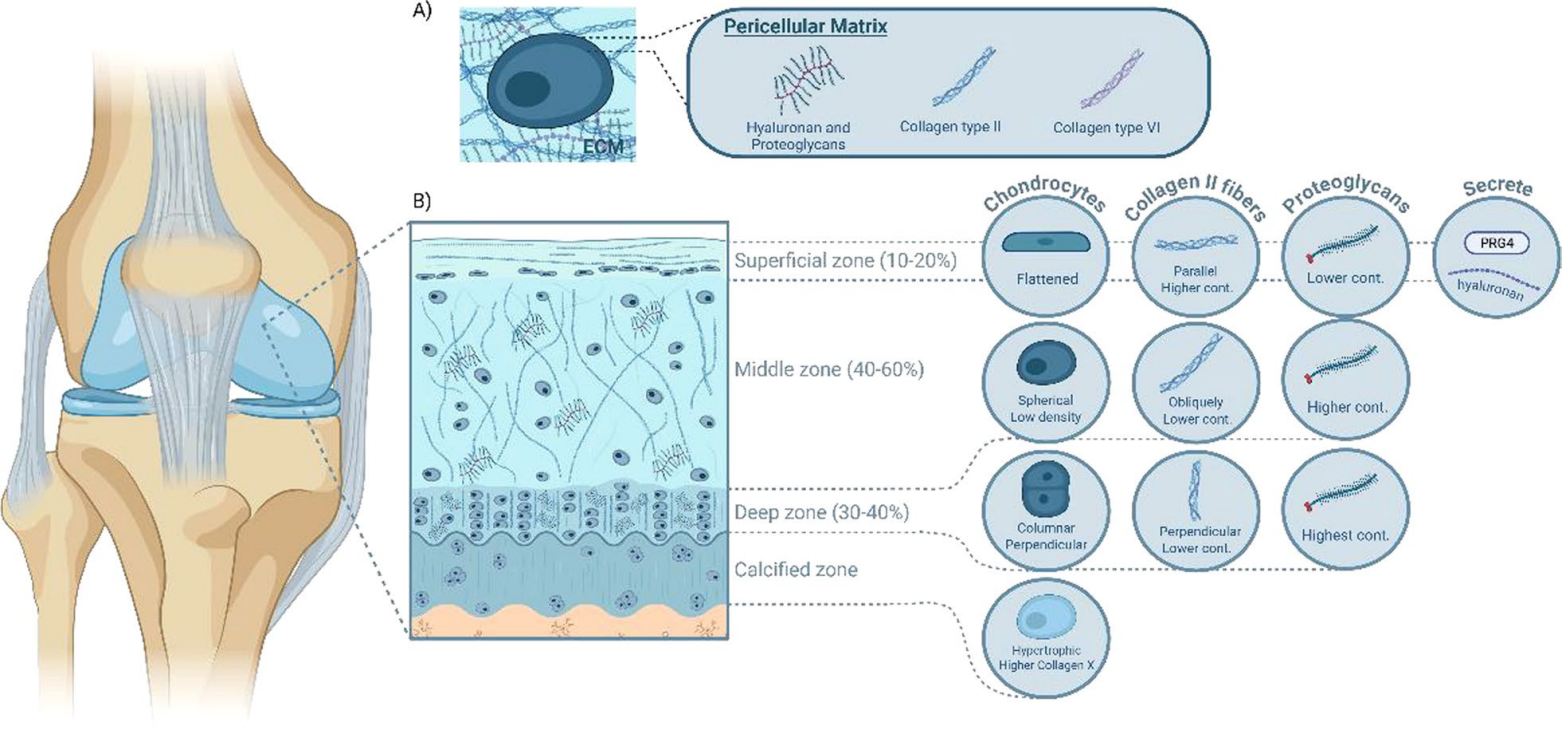
Articular cartilage composition and structure.** A** Schematic of a chondrocyte within its pericellular matrix (PCM), rich in proteoglycans, collagen type II, and collagen type VI, embedded in the extracellular matrix. **B** Zonal structure of articular cartilage: superficial, middle, deep, and calcified zones, showing changes in collagen fibre orientation, proteoglycan content, and chondrocyte morphology across zones

Articular cartilage (AC), a type of hyaline cartilage, is a low-friction, load-bearing connective tissue that covers the ends of bones in diarthrodial joints. It provides a smooth, lubricated surface to absorb and distribute mechanical stress between adjacent bones. AC consists of a stiff, highly hydrated ECM (0.5–4 MPa), primarily composed of negatively charged proteoglycans (e.g., aggrecan) (PGs) and a type II collagen fibril network, along with cells such as chondrocytes. Notably, ECM components support mechanical load and facilitate interstitial fluid pressurization during loading [154]. Chondrocytes (Fig. 1A) are surrounded by a soft pericellular matrix (PCM) (2–25 kPa), which lies between the chondrocytes and the ECM. The PCM primarily contains proteoglycans, type II collagen fibres, and type VI collagen, and it plays a key role in maintaining the chondrocyte phenotype [164]. The thickness of the PCM begins at approximately 2 µm near the cartilage surface and increases with depth, reaching around 5 µm in the deeper zones [337]. Chondrocytes are responsible for synthetizing and maintaining AC constituents [220]. Additionally, AC is hypocellular, lacking blood vessels, nerves, and lymphatic vessels, which impairs its healing potential.

AC is a well-organized tissue that can be divided into four distinct regions **(**Fig. 1B**)**: the superficial zone (SZ), middle zone (MZ), deep zone (DZ), and calcified zone (CZ) [126]. The SZ is thin, accounting for 10–20% of the total thickness of the AC. The chondrocytes in the SZ are flattened and oriented parallel to the AC surface. The type II collagen fibres are abundant and tightly packed, and like the chondrocytes, are parallel to the AC surface, whereas the proteoglycan content is lower. The SZ is in direct contact with the synovial fluid, and the SZ chondrocytes, along with synoviocytes (i.e., synovial-derived cells), produce and secrete lubricating biomolecules (e.g., hyaluronan and proteoglycan-4 (PRG4)) to maintain a highly lubricated surface [75, 132, 270]. The MZ, which accounts for 40—60% of the thickness of the AC, has a higher concentration of proteoglycans but a lower content of thicker type II collagen fibres, which are arranged obliquely, compared to the SZ. The MZ chondrocytes are spherical and at low density. The DZ, which constitutes 30–40% of the AC thickness, contains type II collagen fibres oriented perpendicularly to the AC surface, in lower amounts, while the proteoglycan content is the highest. This zone provides the maximum resistance to mechanical loads. The DZ chondrocytes are columnar and elongated, oriented perpendicular to the AC surface. The final zone, the CZ, is composed of type X collagen, sodium hyaluronate, nanohydroxyapatite, and sparse, hypertrophic chondrocytes. Of note, hypertrophic chondrocytes are enlarged cells that specifically express Col10a1 and metalloproteinase-13 (MMP13) [130].

## Chondrogenic cell mechanotransduction

### a. Cell–matrix adhesion

#### b. Focal Adhesion – mediated extracellular cues transduction

**Fig. 2:**
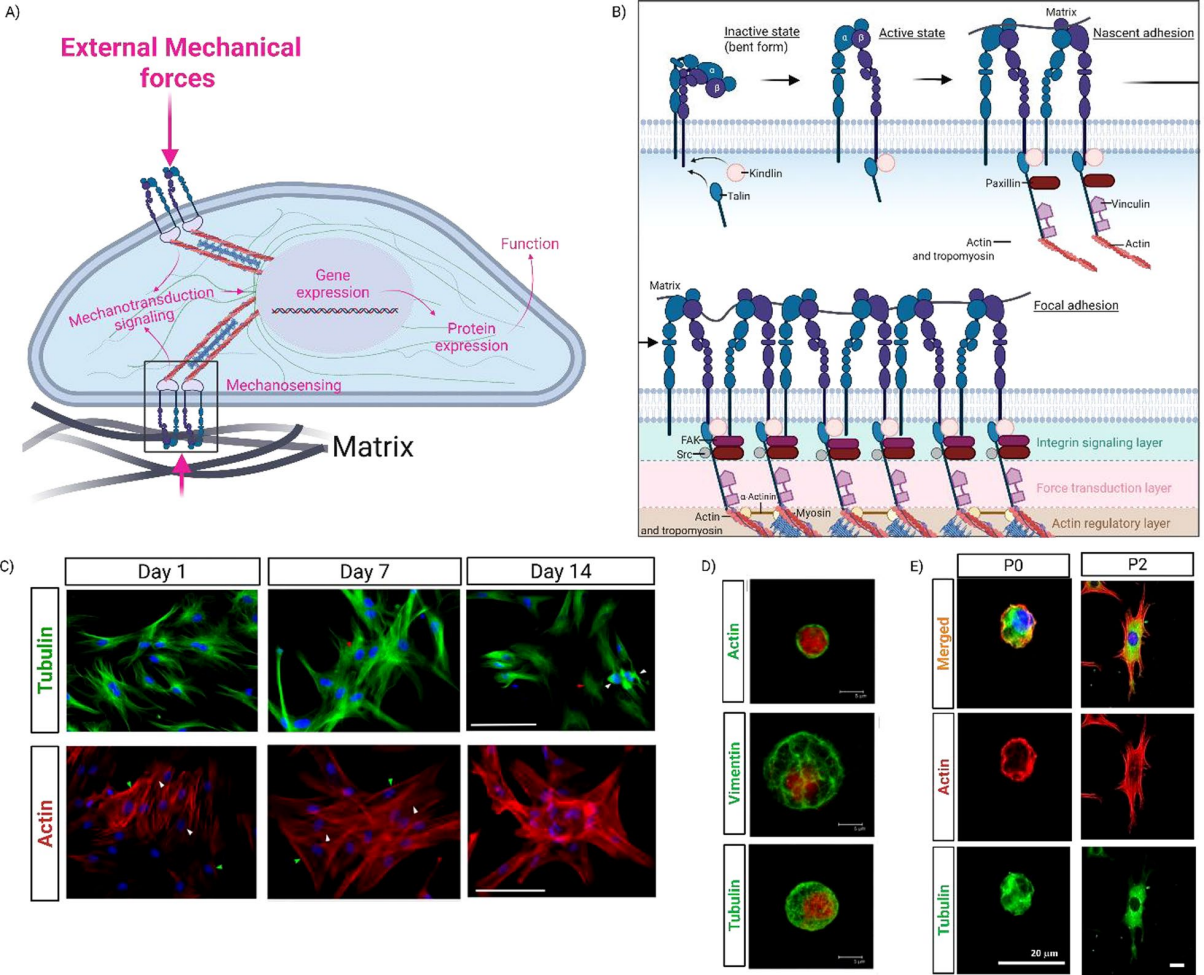
Chondrogenic mechanotransduction.** A** Chondrogenic cells sense mechanical forces and matrix properties through the integrin–focal adhesion complex, which converts these cues into chemical signals that regulate gene expression and protein synthesis, ultimately influencing chondrogenesis and ECM production. **B** Activation, and complex formation of integrin heterodimers. **C** Adapted image from Tvorogova et al., illustrating cytoskeletal organization changes during chondrogenic differentiation of bovine MSCs cultured on a glass monolayer [1]. **D** Adapted image from Blain et al., showing the cytoskeleton of bovine articular chondrocytes after in monolayer culture [24]. **E** Adapted image from Parreno et al., depicting cytoskeletal rearrangements between differentiated and dedifferentiated bovine articular chondrocytes in monolayer culture [242, 244]

Adhesion of chondrogenic cells to the surrounding matrix (e.g. ECM/PCM) **(**Fig. 2A**)** composed of fibronectin, collagen and laminin) is mediated by integrins, which act as transducer of physical cues [287]. Integrins are composed of transmembrane heterodimers of α and β subunits. There are 8 β subunits and 18 α subunits in vertebrates, which assemble into 24 distinct integrin subtypes [127]. Most often, the integrins (Fig. 2B) are in the inactive conformation, which is characterized by “bent” extracellular domains that mask the ECM-binding pocket. When talin and kindlin are recruited, they switch the β-subunit to an active conformation, unmasking the ligand-binding site and enabling integrin–ECM binding. Next, vinculin and paxillin are mobilized making it the Nascent adhesion complex (< 1 um long), which contains only few (3—6) integrin dimers. Subsequently, nascent adhesions mature into larger, more stable focal adhesions (1–5 μm) through clustering of active integrins and recruitment of FAK and α-actinin [39]. Additionally, focal adhesions have a 3D multi-layer structure and are stratified into three functional layers. Near the plasma membrane is the integrin signalling layer, where signalling-related proteins, such as kindlin, FAK, paxillin, non-receptor tyrosine kinase (Src) are localized with integrin cytoplasmic tails. Next is the force transduction layer, in which vinculin resides to connect integrin to actin and thus transmit forces. Far from the plasma membrane is the actin regulatory layer, in which actin-binding proteins such as α-actinin and zyxin localize to regulate F-actin dynamics. Talin spans all three layers, forming the backbone structure of the focal adhesion complex [211].

- Integrins

Human chondrocytes and MSCs express α5β1 (to bind fibronectin), αvβ3 (to bind vitronectin), α6β1 and α3β1 (to bind laminin), α1β1 and α2β1 (to bind collagen I, II and VI) [98, 189]. Interestingly, α5β1 is the most prominently expressed integrin in human AChs and MSC [40, 135] and has been shown to be involved in chondrocyte survival and cartilage matrix degradation in monolayer culture [76, 252]. Additionally, both αvβ3 and β1 integrins have been reported to be involved in the transmission of dynamic compression in bovine chondrocytes embedded in 3D agarose constructs [41]. Supporting this finding, another study by Xu et al. documented that β1 integrin activation by dynamic compression enhances chondrogenic gene expression in chondroprogenitor cells (ATDC5) seeded in a heterogeneous gelatin methacrylate (GelMA) construct [333]. For MSC chondrogenesis, the integrin pathway also contributes to mediating external physical stimuli, yet its role is unclear. For instances, Zhang et al. showed dynamic compression downregulates β1 integrin–FAK–ERK signalling in human Wharton’s Jelly MSCs on chitosan-coated poly(L-lactide-co-ɛ-caprolactone) (PLCL) scaffolds, inhibiting hypertrophic differentiation while promoting TGFβ–SMAD2/3 signalling [345, 346]. Remya et al. reported β1 integrin activation mediates the enhancement of chondrogenesis by dynamic compression in hBMSCs (human Bone Marrow stromal cells) seeded in Poly (vinyl alcohol)-poly(caprolactone) (PVA/PCL) scaffolds [258]. Altogether, these results suggests that the cellular response to dynamic compression might be context-dependent and influenced by the scaffold material. Nevertheless, β1 integrin appears to be a critical regulator of the chondrogenic response to dynamic compression for both MSC and Ch.

- FAK and Src

FAK is a non-receptor tyrosine kinase that controls focal adhesion dynamics and is indirectly anchored to the β subunit of integrins through talin and paxillin [212]. FAK consist of three domains including an N terminal FERM domain, a central catalytic tyrosine kinase domain, and a C-terminal FAT domain [192]. The activation of FAK, which is regulated by growth factors and/or integrins, is characterized by autophosphorylation at a tyrosine residue (i.e., Tyr-397), displaying a high affinity binding site for Src [241]. Upon binding to Tyr-397, Src phosphorylates two tyrosine residues (Tyr-576 and Tyr-577) in the FAK kinase activation loop, forming an active FAK-Src complex [36]. This active FAK-Src complex stimulates signalling pathways, such as PI3K/Akt, MEK/ERK, p38 and RhoA/ROCK. Src kinase has been shown to negatively regulate the chondrocyte phenotype, as its inhibition promotes chondrogenic gene expression, induces cell rounding, and reduces stress fibre formation in primary mouse chondrocytes cultured in monolayer, according to Bursell et al. study [35]. Moreover, FAK has exhibited phase-dependent roles in MSC chondrogenesis. It promotes early condensation in chick mesenchymal cells [15], inhibits subsequent chondrogenic differentiation in mouse mesenchymal stem cells [238], and in hBMSCs, its inhibition prevents hypertrophy [345, 346]. For chondrocytic cells, studies have shown that FAK is essential for mechanotransduction. Evidence includes reduced GAG secretion in monolayer-cultured human chondrosarcoma (SW1353) cells following FAK silencing [241], periodic perfusion-induced FAK activation in monolayer-cultured rat ACh promotes proliferation and matrix synthesis [181], and FAK-dependent upregulation of chondrogenic gene expression in dynamically compressed chondroprogenitor cells (ATDC5) embedded in heterogeneous GelMA constructs [333]. Furthermore, inhibiting FAK prevents rat AChs from dedifferentiating on fibronectin-coated surfaces [279].

- Talin

Talin is a mechanosensitive scaffold protein that directly binds to the cytoplasmic domain of integrins, activates integrins in conjunction with kindlin, and links integrins to actin filaments [93]. According to the literature, talin has been reported to regulate the chondrogenic phenotype in bovine articular chondrocytes under conditions that promote this phenotype (e.g., suspension culture and BMP-7 supplementation) [304]. It has also been shown to mediate αV integrin–dependent TGF-β activation induced by fluid shear stress in SV40-immortalized human chondrocytes cultured in monolayer [350].

- Kindlin

Kindlin is also a scaffold protein involved in inside-out integrin activation by direct binding to the cytoplasmic domain of integrins. In mammals, the Kindlin family consists of three members: Kindlin-1, −2, and −3. [325] demonstrated that Kindlin-2 is crucial for skeletal development. Conditional ablation of Kindlin-2 in limb mesenchymal progenitors in mice (*Kindlin-2*^*Prx1*^ cKO) showed decreased chondrocyte proliferation, increased apoptosis, increased chondrocyte hypertrophy, and disrupted chondrocyte column formation [325]. Another study demonstrated that Kindlin-2 controls MSC commitment to different cell lineages, whereas depletion of Kindlin-2 promotes adipogenesis and inhibits osteogenesis, both in a Kindlin-2 -knockout mice (*Kindlin-2*^*Prx1*^ cKO) and monolayer cultured Kindlin-2 -knockdown MSCs [99]. Altogether, both studies suggest that Kindlin -2 supports skeletal development and acts as a key regulator of mesenchymal stem cell fate. With respect of Kindlin-3, Kerr et al. study has shown it may play an opposing role in chondrogenesis compared to Kindlin-2, acting as a negative regulator and modulating chondrocyte hypertrophy [143]. Nonetheless, although the role of Kindlin in MSC chondrogenesis and skeletal development has been studied, the function of both Kindlins in responding to mechanical stimuli in chondrogenic cells has not yet been elucidated.

- Vinculin and Zyxin

Vinculin is an actin-binding protein (ABP) that is part of the focal adhesion complex. Koshimizu et al. demonstrated that vinculin regulates chondrogenic maturation in primary mouse chondrocytes [152]. Moreover, Steward et al. also observed that vinculin expression increases during chondrogenesis under mechanical loading in porcine BMSCs cultured in agarose constructs, indicating a potential role in mediating mechanotransduction during this process [284]. Another component of the focal adhesion complex is Zyxin. Li et al. demonstrated that knockdown of zyxin in rabbit primary chondrocytes cultured in monolayer led to reduced expression of actin cytoskeleton and vinculin, as well as COL II and COL X production, suggesting that zyxin can ultimately regulate collagen synthesis [170].

Herein, we summarize the key findings on the role of focal adhesion structures in mechano-chondrogenic responses and highlight current limitations identified in the literature:

- β1 integrin signalling is involved in chondrogenic responses to dynamic compression in hMSCs [258, 345, 346] and bovine chondrocytes [41], but reported effects are controversial.
- FAK activation mediates the pro-chondrogenic effects of mechanical stimuli, such as perfusion stress in rat AChs [181] and dynamic compression in chondroprogenitor cells (ATDC5) [333].
- Talin is essential for fluid shear stress-induced TGFβ activation mediated by αv integrin in monolayer-cultured immortalized human chondrocytes (SV40) [350].
- Kindlin-2 plays a pro-chondrogenic role [99, 325], whereas Kindlin-3 supports hypertrophic chondrogenic development [143]. To date, the role of both Kindlins in mechanically induced chondrogenesis remains unexplored.
- Vinculin and Zyxin control chondrogenic maturation [152] and collagen production [170] respectively. Vinculin is also involved in the response to dynamic compression stimuli [284].

### c. Cytoskeleton

The cytoskeleton, a complex three-dimensional network, confers structural integrity to cells and modulates essential biological function (e.g., proliferation and fate). Consisting of cell microfilaments (MFs) (i.e., actin cytoskeleton), microtubules (MTs) (i.e., tubulin) and intermediate filaments (IFs) (i.e., vimentin-cytoplasm IFs and lamin-nuclear IFs), each element of the cytoskeleton network performs unique mechanical roles. Among the cytoskeletal filaments, only MFs and MTs are polar, characterized by their ability to polymerize at one end while depolymerizing at the other.

- Microfilaments

MFs often described as the backbone of the cytoskeleton, are essential for maintaining cell shape, driving motility, supporting division, and facilitating intracellular transport. MFs, also known as actin filaments (approximately 7 nm in diameter), are composed of two linear polymers of Globular(G)/monomer-actin twisted around each other, along with regulatory proteins such as troponin and tropomyosin (TPM) [306]. The formation of MFs is a dynamic process in mammalian cells, involving cycles of polymerization of G-actin into F-actin and depolymerization (the reverse process) [234]. Linear arrays of those actin filaments crosslinked by actin-bundling proteins (e.g., actinin, fascin, and filamin) are known as F-actin bundles [278]. F-actin bundles, containing approximately 10–30 thin filaments (F-actin), interleave with thick filaments composed of non-muscle myosin II (NMMII), forming structures known as stress fibres [303]. Of note, in addition to these actin-bundling proteins, there are also actin-severing and capping proteins, such as adseverin, which has been shown to regulate the bovine ACh phenotype through modulation of the actin cytoskeleton [42]. The actin cytoskeleton in hBMSCs (Fig. 2C) cultured in a monolayer consists of elongated and parallel stress fibres that extend across the entire cytoplasm [282]. Throughout chondrogenesis, the actin cytoskeleton transforms into short thin actin bundles chaotically distributed across the cytoplasm (A. Tvorogova, V. Kovaleva, and A. Saidova 2018). In freshly isolated primary bovine AChs (Fig. 2D and E), the actin cytoskeleton is cortically arranged and maintains a high G-/F-actin ratio, whereas dedifferentiated chondrocytes (i.e., passaged chondrocytes cultured in monolayer) exhibit prominent actin stress fibres and a significantly lower G-/F-actin ratio [24, 242, 244]. Altogether, these observed variations in actin cytoskeleton structure suggest that MFs may play a key role in regulating stem cell fate [48, 204, 338]. For instance, in two studies, actin disruption promoted chondrogenesis in chick MSCs cultured in monolayer [147, 183]. Other studies using AChs, reported actin cytoskeletal depolymerization either by simvastatin [105] or via cytochalasin D [228, 242, 244] promoted better chondrogenic performance. Additionally, Parrero et al. showed that passaged bovine chondrocytes in monolayer culture were prevented from dedifferentiating when exposed to an actin depolymerization agent [117, 242, 244]. Furthermore, studies have shown that external mechanical forces can regulate the actin cytoskeleton. For example, Zhang et al. reported that dynamic compression applied to hBMSCs seeded on a PLCL/chitosan scaffold promoted actin depolymerization and a rounded cell morphology with peripheral localization of the actin cytoskeleton. In contrast, hBMSCs in the free-swelling scaffold displayed a more spread morphology with polarized F-actin stress fibres [345, 346]. Additionally, another study applying dynamic compression to hBMSCs in collagen constructs reported the formation of novel actin protrusions, termed mechanopodia, which were induced by the applied loading [114].

- Intermediate filaments

While MFs are recognized as tension sensors, IFs are mainly involved in absorbing mechanical stress. Furthermore, IFs play additional roles, including protecting cell architecture and nuclear integrity, supporting the positioning of organelles within the cytoplasm, and facilitating crosstalk between MFs and MTs. Vimentin (8—12 nm of diameter), the dominant type of IFs presents both in MSCs and chondrocytes, is involved in migration and differentiation such as chondrogenesis [28, 71]. Moreover, a study reported that the vimentin network morphology in MSCs and chondrocytes is comparable in the perinuclear region. However, in the peripheral region, MSCs exhibit a denser network, whereas chondrocytes display a lower network density [193]. In the case of uniaxial compression stimuli, a study demonstrated that a deficient vimentin network enhances resistance to deformation of hBMSCs encapsulated in agarose gels. The study further investigated the outcome and concluded that the observed behaviour may be due to actin filament rearrangement compensating for changes in vimentin network [275].

- Microtubules

MTs or tubulin facilitates directional transport across the cytoplasm by generating push–pull forces and plays a key role in resisting compressive stress. Tvorogova et al. reported that during bovine MSC chondrogenesis in monolayer, microtubules undergo reorganization, becoming non-radial and non-centrosomal, and orienting transversely to the cell radius. This rearrangement ultimately results in the formation of parallel thick MT bundles and curved MTs surrounding the nucleus (A. Tvorogova, V. Kovaleva, and A. Saidova 2018). Furthermore, a study showed that stabilizing microtubules (i.e., tubulin) enhanced chondrogenesis in synovial hMSCs cultured as pellet [171, 176]. Another study demonstrated that the tubulin network and vimentin remained intact following dynamic compression, whereas the actin cytoskeleton collapsed in response to loading in hBMSCs encapsulated in collagen constructs [114]. In bovine AChs, the polymerization status of tubulin appears to remain stable throughout passaging, indicating that microtubules may not be directly involved in the dedifferentiation process [242, 244].

As a summary of this section, we outline the key findings on cytoskeletal structures in mechano-chondrogenic responses, note the current limitations, and suggest future research directions:

- Actin cytoskeleton organization in hBMSCs is influenced by external mechanical forces such as dynamic compression, resulting in cortical actin arrangement and F actin depolymerization [345, 346]. Actin depolymerization enhances chondrogenic performance in MSCs [147, 183], AChs [105, 228, 242, 244], as well as dedifferentiated AChs [117, 242, 244].
- While actin filaments play the primary role in resisting cell deformation under compression in hBMSCs [275], vimentin and tubulin can compensate when the mechanically induced collapse of the actin cytoskeleton occurs [114]. This highlights the cooperative interplay among cytoskeletal components in maintaining cellular integrity. Regarding the role of vimentin and tubulin in the chondrogenic differentiation of MSCs and AChs, further research is needed.

### d. Chondrogenic and mechanically related molecular pathways

#### e. TGF-β and TGF-β signalling pathway

TGF-β is a key cytokine that plays vital roles in various cellular processes, such proliferation, differentiation, matrix formation and inflammation. Also called Cartilage-Inducing factor, this cytokine is required for chondrogenesis of human BMSCs [16, 273]. In mammalian tissue, three peptides are identified TGF-β1, TGF-β2 and TGF-β3. All three peptides are highly expressed during skeletal development, exhibiting overlapping yet distinct spatial and temporal expression patterns [260]. Moreover, although these three peptides are highly homologous, knockout studies in mice reveal non-overlapping in vivo functions across them. TGF-β1 knockout mice exhibit severe inflammation resembling an autoimmune disorder [280]. Mice deficient in TGF-β2 show several morphologic defects during development [265]. TGF-β3 deficiency in mice results in lung developmental abnormalities and cleft palate [138]. In vitro chondrogenesis of hBMSCs showed no significant differences among the three TGF-β subtypes in cartilage matrix production, although TGF-β2 demonstrated the ability to produce more ECM than the other subtypes [37]. The biological activities of the three TGF-β isoforms are regulated through three key processes: synthesis, latency, and activation.

- TGF-β synthesis and latency

**Fig. 3:**
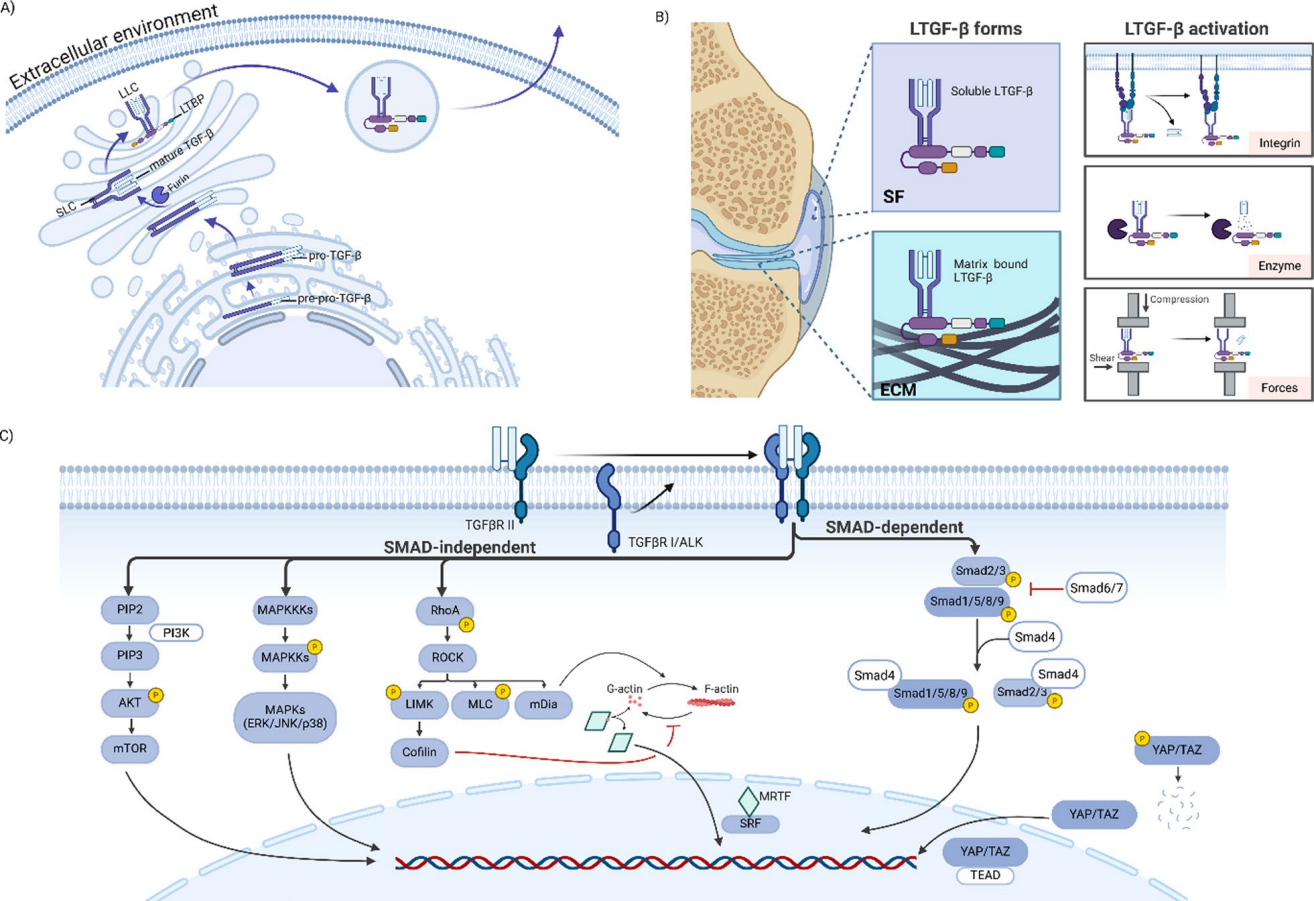
Key chondrogenic and mechanotransduction cellular processes **A** TGF-β Biosynthesis. TGF-β is synthesized as pre-pro-TGF-β in the endoplasmic reticulum, where it loses its signal peptide and dimerizes to form pro-TGF-β. In the Golgi, furin cleaves pro-TGF-β into mature TGF-β and latency-associated peptides (LAPs), forming the small latent complex (SLC). The SLC then binds to latent TGF-β binding protein (LTBP), generating the large latent complex (LLC) for secretion. **B** Latent TGF-β localization and activation. **C** SMAD-dependent and SMAD-independent signalling pathways.

TGF-β (Fig. 3A) is produced by various cell types, such as MSCs and chondrocytes [289], and functions as an autocrine biosynthetic mediator [263]. Initially, pre-pro-TGF-β is synthesized, comprising three regions: the N-terminal signal peptide (SP), the latent-associated peptide (LAP), and the C-terminal segment. In the endoplasmic reticulum, the SP is removed, and the remaining portion of pre-pro-TGF-β dimerizes through disulfide bonds to form pro-TGF-β. The pro-TGF-β then transits to the Golgi, where it is cleaved by the protease furin into mature cytokine segments and LAP, ultimately forming a small latent complex (SLC) through non-covalent interactions. Subsequently, the SLC associates with the latent TGF-β binding protein (LTBP) through disulfide bonds, forming the large latent complex (LLC), which is then transported from the Golgi for secretion from the cell [131]. Once secreted from the cell, LTGF-β (Fig. 3B) can exist in a soluble form (e.g., in culture media) or a matrix-bound form. For example, in the synovial joint, LTGF-β can be present in the synovial fluid as a soluble form (in the range 1—10 ng/mL) of [4] or bound to the articular cartilage ECM components (up to approximately 300 ng/mL) [214]. Furthermore, it has been demonstrated in other tissues that LTGF-β, via LTBP, can bind to fibronectin, fibrillin [46] and elastin [140]. Tang et al. reported connective tissue growth factor (CTGF) as another LTGF-β binding protein that binds to heparan sulphate chains of perlecan in the pericellular matrix of chondrocytes [286].

- TGF-β activation

While LTGF-β is present in considerable amounts, either in soluble form or bound to the ECM, the activation levels were found to be relatively low, ranging from 0.01 to 0.7 ng/mL in the synovial fluid and AC [4–6]. The activation of latent TGF-β occurs through the release of the mature TGF-β from the large latent complex (LLC). This process can be mediated by various factors, including integrins, proteases and external mechanical forces.

αv integrins, such as αvβ3, αvβ5, and αvβ6, have been shown to be involved in the activation of articular cartilage ECM-bound latent TGF-β by binding to the RGD sequence present in the LAP of TGF-β1, TGF-β2, and TGF-β3 [159, 350]. This binding induces a conformational change in the LLC, leading to the release of the mature cytokine.

In addition to integrin-mediated latent TGF-β activation, external mechanical forces, such as fluid shear stress, dynamic compression alone or in combination with shear, have been shown to activate both LTGF-β soluble form [4, 82, 83] and ECM-bound LTGF-β [82, 83, 196]. Recently, Albro's group developed an autoinduction biomarker assay for measuring in situ TGF-β activity, enabling the assessment of the role of dynamic compression in activating ECM-bound latent TGF-β in cartilage explants. Their findings suggest that mechanical load is a key contributor to the activation of ECM-bound latent TGF-β. This conclusion contrasts with earlier work from the same group, which employed a less robust technique [5, 6]. Additionally, in the same study, they demonstrated that the cell-related activation mediators (i.e., the aforementioned) play a lesser role than loading in activation of ECM-bound latent TGF-β [318]. Furthermore, Zhen et al. proposed be a mechanically induced feedback pathway (i.e., outside-in and inside-out) underlying ECM-bound latent TGF-β activation. Specifically, as briefly mentioned in the Talin section, this mechanism involves mechanical stress talin-centered cytoskeletal reorganization, which enhances cell contraction forces and thereby activating αv integrin-mediated latent TGF-β in chondrocytic cells [350].

Additionally, matrix metalloproteinases such as tromelysin-1 (MMP-3) [197] and collagenase-3 (MMP-13) [59], plasmin [246], transglutaminase [261] and lysophospholipids[84] can activate cartilage matrix-bound latent TGF-β.

Next, active TGF-β can bind to the surrounding cell matrix. Studies have shown that active TGF-β binds to multiple ECM structural proteins, such as proteoglycans [112], glycosaminoglycans[113, 194], collagen type II [253] and cartilage oligomeric matrix protein (COMP) [108]. Moreover, active TGF-β transported from the synovial tissue seems to accumulate in the superficial zone (SZ) (0—250 µm deep) and is unable to penetrate deeper into AC due to the presence of an overwhelming concentration of nonspecific TGF-β binding sites in the SZ [5, 6]. Interestingly, Albro et al. more recently observed gradients of active TGF-β in early-stage agarose constructs, which lacked ECM components and were therefore unlikely to bind active TGF-β. In these constructs, TGF-β concentration was highest at the interface and decreased toward the centre, a pattern seen with both bovine ACh- and hMSC-seeded constructs, likely due to high-affinity binding and rapid cellular internalization [7].

Additionally, heterogeneity within the construct, driven by the active TGF-β concentration gradient, was evident, with dense cell clusters forming at the construct periphery compared to the centre. This phenomenon appears to result from the rapid cell proliferation induced by active TGF-β [7]. The authors speculated, based on the resemblance of chondrocyte morphology to hypertrophic chondrocytes in the growth plate, that the peripheral chondrocytes may have undergone a hypertrophic phenotype. Although this was not confirmed in their study, another study reported peripheral deposition of collagen type I in bovine ACh-encapsulated agarose constructs [53]. This observation of peripheric cell clustering led the Albro team to investigate the effect of TGF-β doses on cartilage composition, specifically whether they promoted a hyaline cartilage-like (or chondrogenic) or fibrocartilaginous phenotype. They found that a supraphysiological TGF-β dose ($\ge$ 10 ng/mL) promoted cell cluster formation and increased collagen type I deposition compared to a physiological dose (0.1–1 ng/mL) in bovine AChs seeded in agarose constructs. Therefore, a physiological TGF-β dose has been shown to be a superior supplementation strategy, as it promotes ECM biosynthesis while preventing fibrocartilage formation and hyperplasia-associated dense cell clusters [317].

Activated TGF-β induces multiple intracellular signalling pathways, which are categorized as SMAD-dependent or-independent (Fig. 3C).

### a.1. SMAD-dependent signalling

When activated TGF-β, can initiates SMAD signalling, named after "small mothers against decapentaplegic." This process begins when the ligand binds to the transmembrane serine/threonine kinase receptor TGF-β type II receptor (TGF-βRII). This binding prompts TGF-βRII to recruit and phosphorylate the TGF-β type I receptor (i.e., also termed activin receptor-like kinases (ALKs)), which subsequently propagates the signal through the activation of SMAD proteins. SMADs are classified based on their functions as follow: receptor-regulated SMADs (R-SMADs), which include SMAD1, 2, 3, 5, and 9 (also known as 8) and act as transcription factors (Hill 2016); the common-mediator SMAD4 (Co-SMAD4), which binds to activated R-SMADs and facilitates the nuclear translocation of the complex (Zhao, Mishra, and Deng 2018); and inhibitory SMADs (I-SMADs), such as SMAD6 and SMAD7, which prevent R-SMAD phosphorylation, inhibit R-SMAD DNA binding, or interfere with R-SMAD/Co-SMAD complex formation [357]. Following their activation by TGF-β receptors, the phosphorylated SMADs form a complex with SMAD4 and translocate into the nucleus. Within the nucleus, this complex binds to DNA and modulates gene expression by recruiting transcriptional co-repressors and co-activators. For example, SOX9 (Sex Determining Region Y-Box 9) is an important transcriptional co-activator of chondrocyte phenotype, and its expression directly activates the transcription of chondrogenic genes (e.g., Col2a1 and aggrecan) and through indirect upregulation of Sox5 and Sox6 [80].

Depending on the specific ALKs involved, TGF-β signalling can activate different R-SMADs. Activation of ALK5 leads to the phosphorylation of SMAD2 and 3 [173], whereas activation of ALK1 results in the phosphorylation of SMAD1, 5, and 9 [25, 73]. For decades, ALK5-SMAD2/3 route has been recognized for inducing a stable chondrocyte phenotype, whereas ALK1-SMAD1/5/9 pathway is associated with promoting hypertrophy or terminal differentiation. However, recent studies (described below) challenge these well-established notions [111]. Van der Kraan’s group has shown that both SMAD pathways, as well as ALK5 and ALK1, are required for TGF-β-induced chondrogenic differentiation of BMSCs. Inhibition of either SMAD2/3 or SMAD1/5/9 prevents chondrogenesis in hBMSC pellet culture [111], and a similar effect is observed when ALK5 or ALK1 is knocked down [61]. Interestingly, a recent study has shown that, contrary to the expectation that TGF- β signalling through ALK1 would promote a more hypertrophic phenotype, Diederichs et al. demonstrated that promoting ALK1 does not lead to in vitro hypertrophy and that inhibition of ALK1 was not sufficient to stabilize the chondrogenic phenotype in vivo. This study further demonstrates, for the first time, that ALK4/5 can strongly cross-activate SMAD1/5/9 during chondrogenesis under supraphysiological TGF-β concentrations in hMSC pellet cultured. These findings challenge the conventional view of ALK5-SMAD2/3 as strictly pro-chondrogenic and ALK1-SMAD1/5/9 as solely pro-hypertropic, highlighting the need to elucidate the mechanisms underlying hypertrophy [65].

Regarding the effect of external mechanical forces on SMAD pathways, Bougault et al. demonstrated that dynamic compression activated SMAD2 after only 5 min of stimulation, whereas longer stimulation periods did not result in similarly significant activation in mouse chondrocytes embedded in agarose constructs. This finding suggests that shorter stimulation cycles may be more effective than longer ones, although chondrogenic outcomes need to be assessed to confirm this [30].

### a.2. SMAD-independent signalling

#### a.2.1 Rho-GTPase pathway

The Ras homolog gene family (Rho) of small guanosine triphosphatase (GTPase) are molecular switchers that regulate actin cytoskeleton polymerization, tension and turnover. They cycle between the inactive guanosine diphosphate (GDP)-bound form and the active guanosine triphosphate (GTP)-bound form via guanine nucleotide exchange factors (GEFs), which promote activation, and GTPase-activating proteins (GAPs), which facilitate inactivation. The GDP-bound form remains in the cytoplasm due to its association with guanine nucleotide dissociation inhibitors (GDIs) [232]. The three major Rho-GTPase are: Rho member A (RhoA), Ras-related C3 botulinum toxin substrate 1 (Rac-1) and Cell division control protein 42 (CDC42). RhoA controls stress fibre formation and actomyosin contractile forces, whereas Rac-1 promotes prominent actin structures at the cell periphery known as lamellipodia, and Cdc42 induces the filopodia formation [102]. Additionally, both chondrocytes and MSCs express all three molecules of the Rho-GTPase family [86, 142]. In dedifferentiated or hypertrophic chondrocytes, Rac-1 and Cdc42 activity is higher compared to freshly isolated or immature chondrocytes. Regarding RhoA activity, one study reports enhanced activity [279], while an earlier study suggest it remains unchanged [142]. For clarity, this review will specifically focus on the RhoA/ROCK signalling pathway.

Once RhoA adopts the active GTP-bound conformation, it subsequently activates downstream effectors, such as Rho-associated kinase (ROCK). In mammals, ROCK exist in two isoforms: ROCK1 and ROCK2 [312]. Activated ROCK phosphorylates the myosin-binding subunit (MYPT1), leading to the inhibition of myosin light chain phosphatase (MLCP) and consequently promoting the phosphorylation of myosin light chain (MLC), which supports actomyosin contraction [9, 148]. ROCK also phosphorylates LIM motif-containing protein kinase (LIMK), which in turn phosphorylates the actin-severing protein cofilin, inhibiting its actin depolymerization activity and leading to the stabilization of actin filaments [198]. mDia is another downstream effector whose role is to produce actin filaments through nucleation and polymerization [169].

The RhoA/ROCK pathway is known to regulate chondrogenic potential. Several studies have indicated that MSC chondrogenesis mediated by RhoA/ROCK signalling is highly influenced by the culture model employed. For instances, when cultured on tissue culture plastic (TCP) with RhoA (C3 transferase) or ROCK (Y27632 or ROCKi) inhibitors, rat synovium-derived MSCs show a decrease in Sox9, type II collagen and aggrecan gene expression during chondrogenesis [332]. Furthermore, ROCKi promotes hMSC chondrogenesis in pellet culture [313, 319], while exhibiting little effect on chondrogenic gene expression in hMSC-seeded in hydrogels [88]. Moreover, in chondroprogenitor cells (ATDC5), RhoA overexpression leads to decreased GAG synthesis and reduced Sox9 expression while enhancing stress fibre formation, and these effects are reversed by ROCK inhibition [324]. However, the effects of ROCK inhibition in chondrocytic cells are also context-dependent. While it enhances chondrogenesis in 2D monolayer cultures, it produces atypical outcomes in 3D micromass cultures. In these 3D environments, ROCK inhibition increases Sox9 mRNA levels but paradoxically reduces the expression of its target genes such as Col2a1 and aggrecan. This discrepancy is attributed to delayed Sox9 phosphorylation and reduced expression of the essential transcriptional co-activators L-Sox5 and Sox6, which are required for functional Sox9 activity [323]. In terms of hAChs cultured in monolayer, studies have showed that ROCKi enhanced SOX9 and COL2A1 gene expression [199, 291]. Regarding dedifferentiated chondrocytes, Matsumoto et al. reported that ROCKi maintains the differentiated phenotype of monolayer-cultured hAChs [199]. In contrast, Shin et al. showed that inhibition of RhoA/ROCK had a minimal effect in suppressing dedifferentiation of hAChs cultured in monolayer [279].

With respect of external mechanical forces, it has been shown to impact RhoA/ROCK-dependent chondrogenesis and the chondrogenic phenotype. Haudenschild et al. first demonstrated that dynamic compression induces RhoA activation in hACh-embedded in agarose constructs [109], and in a subsequent study, they showed that dynamic compression stimuli enhanced the nuclear localization of SOX9 through the RhoA/ROCK pathway in hAChs-embedded in alginate construct [107]. Additionally, dynamic compression applied to an AChs-laden poly-d,l-lactic acid/polyethylene glycol/poly-d,l-lactic acid (PEG-PDLLA-DA)/ polycaprolactone-poly(ethylene glycol)-polycaprolactone (PEG-PCL-DA) construct enhanced the activity of both RhoA and ROCKII [331]. Furthermore, Zhao et al. study reported that pellet-cultured rat MSC chondrogenesis under hydrostatic pressure shown to be negatively mediated by RhoA and positively mediated by Rac1[349].

#### a.2.2 MAPK (ERK, JNK and p38) pathway

The mitogen-activated protein kinases (MAPKs) family includes three main subfamilies: extracellular signal regulated kinases (ERKs), c-Jun NH2-terminal kinases (JNK) and p38. The MAPK pathway operate as a series of a series of kinase that start with the activation of MAPK kinase kinase (MAPKKK), which then directly phosphorylates the MAPK kinase (MAPKK), ultimately activates MAPK.

Studies have shown that p38 and ERKs are activated by TGF-β and have been demonstrated to regulate TGF-β-induced BMSCs chondrogenesis, with p38 acting as a positive regulator and ERKs as a negative regulator [172, 179, 180, 195]. In contrast, JNK appears to play a minor role in TGF-β-induced chondrogenic differentiation compared to the other two pathways [239]. Additionally, the regulation of TGF-β-induced chondrogenesis seems to depend on potential crosstalk between the MAPK and SMAD signalling pathways [172, 179, 180]. In pellet cultures of dedifferentiated AChs, activated ERK1/2 have been observed, and inhibition of ERK1/2 leads to redifferentiation of dedifferentiated chondrocytes [313, 319]. Inhibition of the p38 pathway in hACh pellet cultures results in downregulation of chondrogenic gene expression and upregulation of hypertrophic gene expression [250]. Regarding the involvement of the ERK/JNK/p38 pathway in response to biophysical stimuli, Xie et al. demonstrated that dynamic compression applied to an AChs-laden PEG-PDLLA-DA/PEG-PCL-DA construct significantly activates ERK1/2 and p38 [331].

#### a.2.3 PI3K/AKT/mTOR pathway

Phosphoinositide 3-kinases (PI3Ks) represents a family of lipid kinases which when activated converts phosphatidylinositol-4,5-bisphosphate (PIP2) into phosphatidylinositol-3,4,5-trisphosphate (PIP3). This activates further downstream effectors such as protein kinase B (AKT). Phosphorylated AKT can activate mammalian target of rapamycin (mTOR).

Studies reported that inhibition of PI3Ks impaired chondrogenic differentiation of mesenchymal cells cultured either in a micromass fashion or pellet, indicating that PI3K is a positive regulator of MSC chondrogenesis [150, 299]. In chondrocytes, the PI3K/AKT/mTOR pathway appears to negatively regulate apoptosis [50] and autophagy [334].

#### a.2.4 MRTF-SRF signalling (actin-regulated pathways)

Actin can directly regulate gene expression via myocardin related transcription factor (MRTF) A in MSCs and chondrocytes [243, 356]. MRTF is a co-activator of serum response factor (SRF). The MRTF/SRF complex binds to the Carg regulatory sequence (CC[A/T] _6_GG) within the promoter region of target genes to regulate transcription. Additionally, MRTF-A also contains actin-binding motifs and exhibits a stronger affinity for G-actin, which leads to the sequestration of MRTF-A in the cytoplasm of the cell. When G-actin polymerizes into F-actin (e.g., in dedifferentiated chondrocytes), MRTF-A is liberated from G-actin and translocates into the nucleus, activating the MRTF-A/SRF pathway [157].

Inhibition of MRTF-A downregulates the gene expression of contractile (α-SMA and TAGLN) and fibroblastic matrix (COL1 and TNC) markers in bovine passaged chondrocytes, as well as negatively regulates the fibroblast growth factor receptor 1 (FGFR1), enhances adipogenesis and reduces osteogenesis in MSCs [128, 242–244, 356]. Knockdown of SRF or MRTF-A/MRTF-B in C3H10T1/2 mesenchymal stem cells promote commitment to the brown adipocyte lineage as well as attenuates TGF-β signalling while augmenting BMP pathway activity [188].

### f. YAP/TAZ signalling

Yes-associated protein (YAP) and transcriptional co-activator with PDZ-binding motif (TAZ) are homologous transcriptional co-activators that are regulated by the Hippo-dependent pathway or by Hippo-independent pathways such as actomyosin-generated cytoskeletal tension mechanism [69], or by a crosstalk between the two. YAP exists in two forms, YAP1 and YAP2. In Hippo-mediated regulation, activation of the Hippo pathway triggers a series of phosphorylation events mediated by MST1/2 (mammalian Ste20-like kinase 1/2) and LATS1/2 (large tumour suppressor-like kinase 1/2). These kinases phosphorylate YAP/TAZ, leading to its inactivation and subsequent retention in the cytoplasm through binding to the 14—3—3 protein, which promotes its degradation. Upon dephosphorylation, YAP/TAZ is released from the 14—3—3 complex, allowing it to translocate to the nucleus, where it interacts with TEAD transcription factors to regulate the expression of target genes [240].

Studies have found that YAP (i.e., more specifically in this case YAP1) promotes the proliferation of C3H10T1/2 cells (i.e., an MSC model) and chondrocytes via TEAD binding, leading to the direct upregulation of Sox6 [63]. Furthermore, YAP has been reported to negatively regulate MSC chondrogenic differentiation [141] or chondroprogenitor cells (ATDC5) differentiation through activation of the Wnt/β-catenin signalling pathway [335]. Regarding chondrocyte maturation, YAP has been reported to play contradictory roles: Deng et al. found that YAP inhibits maturation and hypertrophy by suppressing Col10a1 via Runx2 [63], whereas Karystinou et al. and Lee et al. reported that YAP promotes the hypertrophic stage [141, 162]. Moreover, to investigate the molecular mechanisms underlying chondroprotective cues in joints, Delve et al. conducted a study on superficial zone chondrocytes. They reported that YAP/TAZ regulates the gene and protein expression of proteoglycan 4 (PRG4) and tenascin C (TNC) [62]. In contrast to YAP, which acts as a negative regulator, TAZ expression gradually increases during chondrogenic differentiation and has been found to positively regulate chondrocyte maturation. Like YAP, TAZ also promotes early chondrocyte proliferation [171, 176]. Mechanistically, TAZ interacts with Sox5 to regulate downstream chondrogenic gene expression and proliferation [171, 176]. However, a recent study reported the opposite, indicating that TAZ also plays a negative regulatory role in chondrogenic differentiation. Additionally, the authors also showed that depletion of both YAP and TAZ is necessary to significantly enhance chondrogenic gene expression, as depletion of either alone is not sufficient to substantially affect chondrogenic performance [103]. This contradictory result may be due to the use of different experimental models, with one study using a mouse model and the other using chondroprogenitor cells (ATDC5) monolayer cultures. This highlights the need for additional studies across diverse species and culture systems to determine which functional outcome is most representative.

Regarding the influence of external mechanical forces on YAP response, two studies have shown that cyclic stretching promotes YAP nuclear translocation in MSCs [69, 145]. Of note, Driscoll et al. observed that YAP signalling pathway requires strain transfer through stress fibres for MSCs to respond to dynamic tensile stretch [69]. However, a contrasting result was observed by Sen et al., who reported that cyclic stretching did not trigger YAP nuclear shuttling in MSCs [272]. Several factors may contribute to this controversy, such as the MSC species, plate coating, stretching system, or protocol used. These confounding factors make it difficult to interpret contradictory findings highlighting the importance of developing standardized culture systems to advance research in this field. With respect to shear forces, Zhong et al. reported that increased fluid shear triggered YAP nuclear translocation in rat BMSCs and AChs. In AChs, higher flow rates led to an increase in fibrocartilaginous markers such as collagen type I and a reduction in collagen type II protein expression, thereby promoting chondrocyte dedifferentiation [353, 354]. Moreover, dynamic compression applied to an AChs-laden PEG-PDLLA-DA/PEG-PCL-DA construct increased YAP activity [331].

Furthermore, in addition to external cues, intracellular signals, such as those originating from the cytoskeleton, appear to influence YAP activity and, consequently, the chondrogenic effect. Microtubule stabilization has been shown to reduce YAP activity and enhance synovial hMSC chondrogenesis [171, 176]. As addressed in the previous sections, upstream molecular pathways control chondrogenic process, such as the RhoA signalling pathway. Hallström et al. showed that RhoA-driven mechanoregulation of chondrogenic maturation is mediated by YAP/TAZ activity and translocation, with increased RhoA signalling reducing chondrogenic gene expression by promoting YAP/TAZ nuclear localization [103].

In summary, we present the key findings on the most robustly supported molecular mechanisms driving mechanically stimulated chondrogenic differentiation, along with current knowledge gaps and potential directions for future research:

- ECM-bound LTGF-β activation is primarily driven by external mechanical forces [318]. The mechanism proposed in the literature involves external mechanical forces inducing talin-dependent cytoskeletal reorganization. This generates tension through αvβ6 integrins, promoting a conformational change in LAP, which binds αv, resulting in the release of active TGF-β [350].
- hBMSC chondrogenesis requires SMAD2/3 and SMAD1/5/9 signalling via ALK5 and ALK1. Activation of ALK1 does not induce hypertrophy, and its inhibition alone is insufficient to stabilize the chondrogenic phenotype in vivo [65]. Further research is now required to identify novel candidates that could serve as targets for potential therapies promoting stable cartilage formation.
- 5 min dynamic compression induces maximal SMAD2 activation in agarose-embedded mouse chondrocytes [30].
- RhoA activity is increased by dynamic compression in hAChs [109, 331], whereas it inhibits chondrogenesis under hydrostatic pressure in rat MSCs [349].
- p38 acts as a positive and ERK1/2 as a negative regulator of BMSC chondrogenesis [172, 179, 180, 195], with both pathways strongly activated by dynamic compression in AChs [331].
- PI3K promotes MSC chondrogenesis [150, 299]
- YAP stimulates chondrocyte proliferation [63] and negatively regulates chondrogenic differentiation [141, 335]. YAP translocates to the nucleus in response to fluid shear stress in both MSCs and AChs. In AChs, higher shear levels are associated with increased nuclear YAP and a dedifferentiated phenotype [353, 354]. Dynamic compression enhances YAP activity in AChs [331].

## In vitro Regenerative rehabilitation

**Fig. 4:**
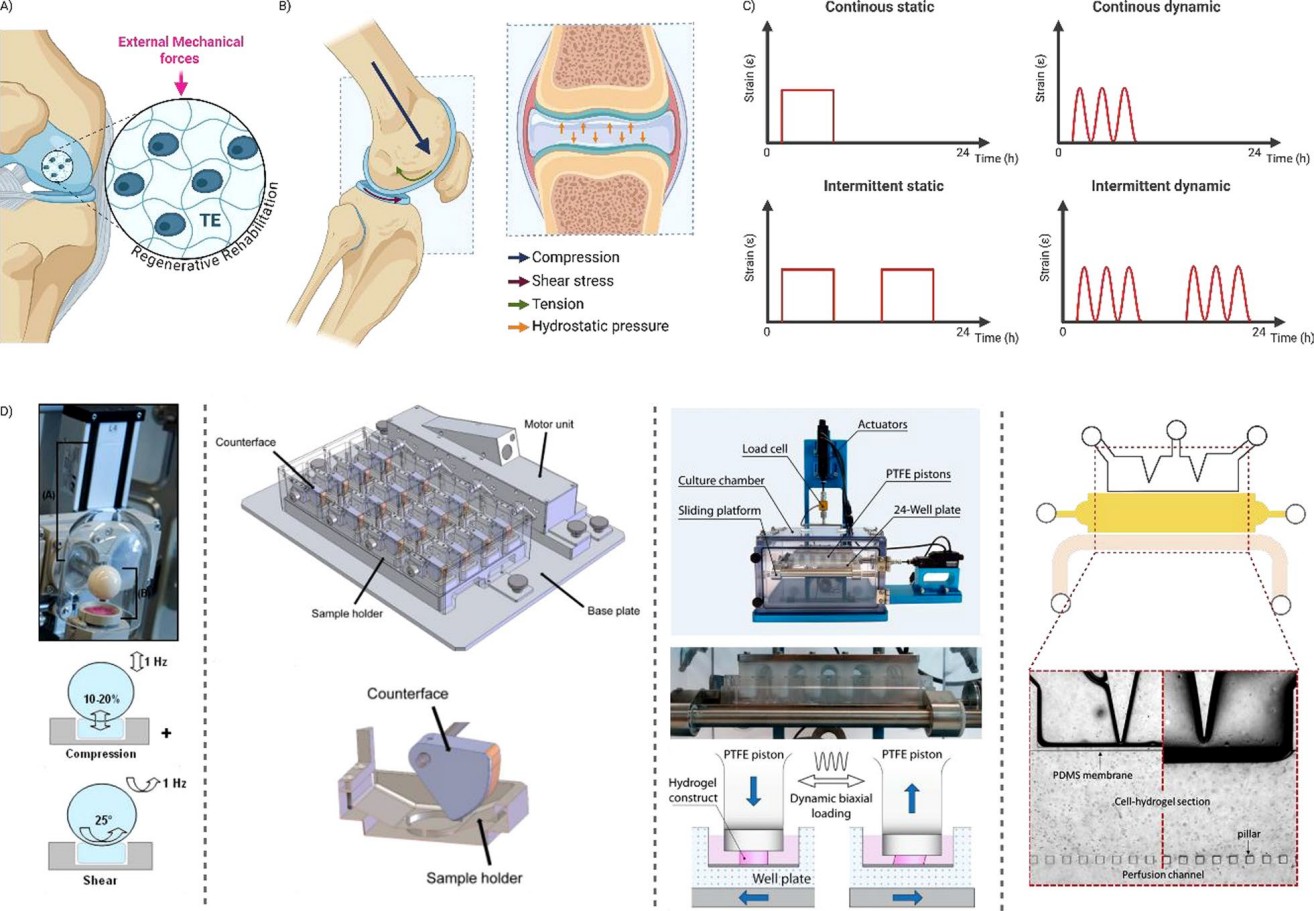
In vitro Regenerative Rehabilitation systems for Cartilage Repair. **A** Schematic illustration of a promising cartilage treatment strategy consisting of a synergistic therapy combining TE with physiotherapeutic rehabilitation routines. **B** Biophysical forces encountered in joints and applied to articular cartilage. **C** Representative waveforms or stress–strain curves describing mechanical loading protocols reported in the literature, as summarized in Tables 1 and 2. **D** Representative summary of commonly used bioreactors and microfluidic devices capable of performing uniaxial and multiaxial compression, as well as shear loading[56, 158, 207, 237]

AC withstands high loads not only during impact sports activities but also during activities of daily living. In the tibiofemoral joint, peak deformation in the contact zone ranged from 7 to 23% of the initial AC thickness during walking [187], while during joint flexion, the deformation peak reached up to 30% of the initial AC thickness [23]. AC is subjected to a range of external biomechanical forces, including compression, shear stress, hydrostatic pressure, and tensile strain, during functional activities such as gait/walking, squatting/joint flexion, and daily living tasks [322]. Abnormal mechanical stresses, such as those resulting from joint trauma, obesity, altered walking kinematics, or changes in knee flexion angles, can initially impact the AC surface. Over time, this may result in cartilage defects that have the potential to progress to osteoarthritis (next section). The prevalence of cartilage defects among patients who have undergone knee arthroscopy is over 60%, with athletic patients exhibiting even higher rates [58, 74]. Current clinically available cartilage repair procedures rely on the bone marrow stimulation (e.g., microfracture) or cartilage restoration techniques (autograft and allograft transplantation and autologous chondrocyte implantation). However, these procedures have certain limitations, such as restricted graft availability from the donor site, mismatched allograft shapes, the formation of fibrocartilaginous tissue, and chondrocyte dedifferentiation during in vitro expansion. Due to these constraints, a promising approach known as cartilage tissue engineering (TE) has emerged in recent decades. This therapy relies on seeding chondrogenic cells onto or into artificial matrices, also known as biomaterials, which are either implanted immediately into the patient or cultured in vitro with the necessary growth factors (e.g., TGF-β, BMPs, and FGF-2) to achieve a hyaline-like cartilage graft before implantation. Following surgery, during the early postoperative period, patients may involve the use a continuous passive motion (CPM) device, which employs an external motorized mechanism to passively move the joint through a specified range of motion [44]. This rehabilitation approach contrasts with the traditional practice of joint immobilization, which has been found to be detrimental to cartilage regeneration and integration. In vivo, static immobilization or reduced joint loading leads to loss of GAG content and decreased PG synthesis, which can be partially rescued by remobilization [18, 137]. In contrast, CPM has been shown to stimulate the production of PRG4 [233]. This synergistic therapy, known as regenerative rehabilitation (RR) (Fig. 4A), combines regenerative medicine (e.g., TE) with physiotherapeutic rehabilitation routines [89, 92]. Clinical evidence in this field remains limited and heterogeneous, restricting the establishment of standardized “gold standard” rehabilitation protocols and resulting in variability in clinical decision making, often guided by surgeon experience [3, 182, 235]. In contrast, in vitro RR research has been investigated more extensively over many years, as discussed in the following section. Some limitations observed in clinical studies, such as variability from confounding factors, are also present in in vitro research, but the larger number of studies may help highlight general trends. At the same time, it is still necessary to summarize these findings in order to establish a consensus on the most appropriate in vitro mechanical stimulation regime to promote cartilage formation. Such a consensus would enhance understanding of effective in vitro RR protocols and support their translation into studies in large animal models and, ultimately, clinical applications. In addition, further studies investigating the interplay between external mechanical stimuli and the intrinsic mechanical cues of TE constructs could provide valuable insights for advancing RR strategies.

## Cartilage tissue engineering constructs

TE constructs have emerged as a promising strategy for cartilage regeneration, as they more closely mimic the three-dimensional native tissue environment. These constructs rely on chondrogenic cells cultured within a 3D or on a 2D a biomaterial designed to replicate the biomechanical properties of cartilage. Essential characteristics of cartilage biomaterials include a controlled biodegradation profile that avoids the release of toxic degradation products, a porous structure to facilitate nutrient and metabolite transport, and the capacity to support cell viability, proliferation, differentiation, and ECM deposition. Moreover, ensure effective integration with the surrounding cartilage defect and provide adequate mechanical strength, given the load-bearing nature of articular cartilage.

### a. Scaffold

#### a.1. Biomaterial classification

Biomaterials can be classified into natural, synthetic, and hybrid. Natural biomaterials are characterized by their high biocompatibility and biodegradability and can be categorized as either protein-based (e.g., collagen type I and II [78, 226], gelatin [12, 248], and silk fibroin [116, 320]) or polysaccharide-based (e.g., hyaluronic acid [185], chitosan [96, 254], alginate [66, 215], and agarose [276]). However, they also present disadvantages, including lower stiffness and limited mechanical properties. Moreover, their predominantly animal-derived origin (i.e., collagen, gelatin, fibrin, silk and Hyaluronic acid) can pose challenges for clinical translation, as these materials may provoke immune responses, display high biological variability, and raise ethical concerns. This has motivated the development of animal-free alternatives. Recombinant protein technologies have been employed to generate non-animal-derived proteins suitable for cartilage tissue engineering[219]. An example of this approach is VECOLLAN®, which consists of recombinant collagen-like proteins assembled into a nonwoven mat using electrospinning [153]. While most natural biomaterials are incorporated into FDA- and EMA-approved medical devices, increased commitment to investment in xeno-free alternatives by both the scientific community and medical device manufacturers is required [55]. Nonetheless, to address their poor mechanical performance, natural biomaterials can be chemically modified or combined with synthetic biomaterials to form hybrid biomaterials. For example, gelatin can be modified with methacrylic anhydride to produce GelMA, which forms a more mechanically stable hydrogel [230]. Additionally, GelMA has been shown to support cartilage-like matrix production by encapsulated chondrocytes and chondrogenesis of MSCs[166–168]. Synthetic biomaterials, such as polylactide (PLA)[262], polyglycolide acid (PGA)[139, 264], polyurethane (PU)[160, 178], polycaprolactone (PCL)[146, 174], poly-lactic-co-glycolic acids (PLGA)[14, 298], polyvinyl alcohol (PVA)[22], and polyethylene glycol (PEG) [34, 259], in contrast to natural biomaterials, offer well-controlled and highly tuneable mechanical properties. The PCL, PLGA, PEG, PGA, PLA are also FDA-approved biomaterials that are commonly in cartilage TE [55, 251].

#### a.2. Biomaterial types

In this review, we focus on sponges and hydrogels, although other biomaterial types such as films, electrospun fibres, bioceramics and bioglass are also widely used. Hydrogels are soft (approximately 1–100 kPa), viscoelastic, water-swollen polymer networks that closely replicate the conditions of cartilage's native ECM and typically possess porosity on the nanometer scale (1–10 nm). However, they generally exhibit insufficient mechanical strength, and their nanometer-scale porosity structure can limit the diffusion of nutrients and waste products. In contrast, sponges are stiffer structures (typically above 100 kPa) with a macroscopic porous architecture that serve as substrates for culturing cells on the top or for seeding within them (for instance, in combination with a hydrogel). For example, Lee et al. demonstrated that combining fibrin hydrogel with a macroscopic polyurethane scaffold significantly improves sGAG retention and markedly upregulates chondrogenic gene expression in bovine ACh [160]. Interestingly, while the Microfilaments section focuses on monolayer cultures, studies using artificial matrices to culture chondrogenic cells (MSCs and chondrocytes) show that the type of matrix influences actin cytoskeleton organization. For instance, studies by Zhang et al. showed that both pig AChs and BMSCs adopted a rounded shape with a cortical actin cytoskeleton when encapsulated in chitosan hydrogels, whereas a spindle-shaped morphology with prominent stress fibre actin cytoskeleton was observed in chitosan sponges [343, 344]. In the case of AChs, these morphological and F-actin cytoskeletal changes were associated with a more fibrocartilaginous and hypertrophic phenotype within sponges, compared to cells encapsulated in hydrogels [344]. This suggests that chitosan hydrogel encapsulation may be a more favourable option for bovine AChs.

In addition to the source or type of biomaterial, properties such as stiffness, porosity, viscoelasticity, cell adhesion ligands, and surface topography may also influence cellular responses and the efficiency of chondrogenesis.

- Stiffness

Stiffness, or elasticity, reflects how much a material resists deformation and is often described as the rigidity of a matrix. Studies on the effect of biomaterial stiffness on MSCs have produced unclear results, which may be influenced by other properties of the biomaterials. For example, Bian et al. reported that higher stiffness (≈53.6 kPa) induced a hypertrophic chondrogenic phenotype in hMSCs, whereas lower stiffness (≈3.5 kPa) promoted a more stable chondrogenic phenotype and enhanced matrix production in hMSCs encapsulated within methacrylated hyaluronic acid (HA) hydrogels [21]. In the study by Ai et al., porcine BMSCs were embedded in methacrylated chondroitin sulfate (CSMA)/GelMA composite hydrogels. The authors reported that stiffness decreased along chondrogenesis, with stiff hydrogels dropping significantly from approximately 33 kPa to 5 kPa and soft hydrogels decreasing from around 8 kPa to 2.55 kPa, highlighting the dynamic nature of stiffness in biodegradable hydrogels. They also showed that stiffer hydrogels altered cell morphology, with cells transitioning from a more spread shape to a rounder form, and promoted superior chondrogenesis compared to softer hydrogels [2]. For chondrocytic cells, studies have shown that stiffer hydrogels favour chondrogenic performance. Xu et al. used chondroprogenitor cells (ATDC5)-seeded in heterogeneous GelMA composite containing two stiffness-like hydrogel phases to resemble the different stiffnesses of the pericellular matrix (PCM) and extracellular matrix (ECM). In this study, the authors found that increasing the stiffness of the surrounding zone/ECM enhanced the expression of chondrogenic markers [333]. Another study by Li et al., reported that bovine AChs embedded in stiff GelMA hydrogels (29.9 kPa) exhibited superior chondrogenic performance, accompanied by a rounded morphology and a cortical actin cytoskeleton, compared to bovine AChs in softer GelMA hydrogels (3.8 kPa) [175, 177]. For hAChs, Levett et al. also observed that stiffer hydrogels (1% w/v methacrylate HA combined with GelMA, 41 kPa) increased aggrecan deposition compared to softer hydrogels (0% w/v methacrylated HA combined with GelMA, 29 kPa) [167, 168].

With respect to the previously mentioned chondrogenic molecular pathways, Allen et al. has repported that the (autocrine) TGF-β-induced SMAD pathway is stifness sensitive while (exogenous) TGF-β-substrate stiffness synergistic response is mediated by the p38 pathway in chondroprogenitor cells (ATDC5) cultured on collagen II–coated polyacrylamide substrates. In the same study, they found that inhibiting ROCK in primary murine chondrocytes and ATDC5 cells decreased Col2α1 expression on soft substrates (0.5 MPa), but increased it on stiff substrates (e.g., plastic), indicating that ROCK activity is essential for the chondrogenic response to soft substrate stiffness [8]. Moreover, the YAP/TAZ pathway is also influenced by matrix stiffness. For instance, hBMSCs cultured on a stiff polyacrylamide substrate in chondrogenic medium (with TGF-β supplementation) showed YAP nuclear compartmentalization [77]. For AChs, Zhong et al. reported that rat AChs cultured on stiff polyacrylamide substrates (40 kPa) exhibit increased YAP nuclear translocation and reduced chondrogenic expression levels compared to those cultured on soft substrates (4 kPa). Furthermore, YAP knockdown in AChs cultured on stiff substrates enhanced the chondrogenic phenotype suggesting that reduced YAP expression is linked to the maintenance of a stable chondrogenic phenotype [353, 354]. Interestingly, “cell mechanical memory” describes a cell’s ability to retain the effects of a previous substrate stiffness even after moving to a new environment. Zhang et al. demonstrated that hMSCs primed on stiff tissue culture plastic (~ 3 GPa) accumulated nuclear YAP, effectively committing them to an osteogenic fate. This effect was reversed using a PEG-MAL hydrogel functionalized with HAVDI peptides, which mimic neighbouring cell signals via N-cadherin ligation (i.e., cell–cell signalling is known to override the cells’ mechanical history). This finding provides a new strategy to control MSC stiffness-dependent behaviour for therapeutic applications [341].

- Cell attachments sites

When the selected biomaterial contains arginine–glycine–aspartate (RGD) motifs, cell adhesion occurs through integrin–biomaterial-mediated interactions [54]. In contrast, when RGD motifs are absent, as in hydrogels based on PEG, alginate or agarose, cells are physically constrained rather than interacting with the biomaterial, leading them to typically adopt a rounded morphology that may support a chondrogenic phenotype. This was observed by Pourmohammadali et al. in bovine AChs-encapsulated agarose hydrogels, which demonstrated increased GAG content [249]. Although agarose lacks RGD motifs, preventing direct cell–hydrogel interactions, Steward et al. demonstrated that porcine BMSCs can interact indirectly with the material by depositing their own pericellular matrix (PCM) during chondrogenic culture. The study reported that stiffer hydrogels (25 kPa) initially suppressed cartilage matrix production compared to softer gels (0.5 kPa). However, the application of hydrostatic pressure restored and enhanced chondrogenesis in the stiffer hydrogels, suggesting that external mechanical cues can override the inhibitory effects of matrix stiffness on MSC fate [283]. In a follow-up study using the same agarose model, dynamic compression enhanced chondrogenic expression in stiff gels compared to soft gels, and this effect was also shown to be mediated by integrin–PCM interactions [284]. In addition to RGD motifs and PCM deposition, other factors can be tuned to improve cell–biomaterial adhesion, although they are not the focus of this review. These include charge, wettability, and topography, with topography discussed in the following section [118].

- Viscoelasticity

Viscoelasticity is a mechanical property in which biomaterials exhibit both elastic behaviour, allowing them to recover their shape, and viscous behaviour, enabling energy dissipation during deformation. This viscoelastic behaviour is often characterized by creep and stress relaxation [326, 327]. Huang et al. demonstrated that rabbit BMSCs encapsulated in slower-relaxing collagen hydrogels exhibited enhanced early chondrogenic differentiation, whereas faster-relaxing collagen hydrogels favoured long-term chondrogenesis (both hydrogels differing in viscoelasticity while maintaining the same elastic modulus, i.e., stiffness). This difference, depending on the stage of chondrogenesis, was further assessed and attributed to increased apoptosis and ROCK-dependent myosin hyperactivation in cells encapsulated within slower-relaxing hydrogels compared with those in faster-relaxing hydrogels. Regarding cell morphology and actin cytoskeleton organization, MSCs encapsulated in slower-relaxing hydrogels during early chondrogenesis displayed a rounded morphology with cortical actin localization, whereas those in faster-relaxing hydrogels exhibited a more spread shape with pronounced stress fibres. During long-term chondrogenesis, cells in faster-relaxing hydrogels transitioned to a spherical morphology with cortical actin organization, consistent with their superior chondrogenic performance. Moreover, inhibition of ROCK during long-term chondrogenesis to prevent myosin hyperactivation caused MSCs in slower-relaxing hydrogels to maintain a spherical, cortical actin-based morphology—resembling cells in faster-relaxing hydrogels—which was associated with enhanced chondrogenic gene expression, type II collagen deposition, and an increased GAG/DNA ratio [123, 124]. Another study showed that higher loss tangent (tan(δ)) values, which indicate faster stress relaxation (i.e., a more viscous character), promote hMSC chondrogenesis compared to lower tan(δ) (more elastic matrices). Although the more viscous nature of polyethylene glycol maleimide (PEG-MAL) hydrogels induces rounded cells, it also results in lower cytoskeletal tension and cytoplasmic YAP, whereas more elastic hydrogels exhibit higher cytoskeletal tension and nuclear YAP [311]. These findings reinforce the link between cell morphology and chondrogenic outcomes, highlighting that a spherical shape promotes better chondrogenesis.

- Porosity

Scaffolds with pores in the 200–300 μm range are typically considered the standard for cartilage tissue engineering constructs [101, 106]. Scaffold porosity not only facilitates nutrient transport, signalling, and cell migration but also modulates fluid shear forces on encapsulated cells when TE constructs are implanted in articular cartilage defects. For instance, Melchels et al., by comparing the fluid flow profile generated in silico with the distribution of hAChs seeded in photo-polymerizable poly(D,L-lactide) (PDLLA) scaffolds in vitro, showed that the highest concentration of cells was found in regions where fluid velocity and shear rates were greatest [208]. Moreover, Azizi et al. employed a purely in silico fluid–structure interaction (FSI) model to investigate MSC-seeded oxidized alginate–gelatin (ADA-GEL) hydrogels and reported that a porosity of 38% (i.e., which means the percentage of total void space) promoted cartilage differentiation compared to the other porosities [13].

- Topography

Topography refers to the micro- and nanoscale surface architecture of biomaterials, including roughness and patterns engineered patterns such as grooves, gratings, aligned fibres, pillars, pits, holes, and tubular structures [209]. Wu et al. demonstrated that hBMSCs seeded on nanotopographical PCL in the form of nano-pillars, which induced a round morphology, showed enhanced stable chondrogenic gene expression and cartilage-like matrix production. In contrast, MSCs on nano-holes exhibited a hypertrophic chondrogenic gene expression profile, accompanied by a more polygonal morphology. Nano-gratings, on the other hand, promoted a spindle-shaped morphology and a more fibrous chondrogenic phenotype [328]. A more recent study from the same group, combining both substrate stiffness (PCL—soft, PLA—medium, and PGA—stiff) and topography (nano-pillars and nano-gratings), demonstrated that the pillar surface yielded superior hBMSC chondrogenesis compared to the grating surface. For the pillar surface, the softer substrate promoted a more stable chondrogenic phenotype, whereas the stiffer substrate favoured a hypertrophic chondrogenic phenotype. Notably, MSC morphology was sensitive to substrate stiffness only on the pillar surface, with cells exhibiting a round morphology on the softer material and a polygonal morphology on the stiffer material, while the grating surface induced a spindle-shaped morphology independent of stiffness [329].

### b. Chondrogenic cell sources

Besides the scaffold, another crucial component of the engineered cartilage constructs is the source of chondrogenic cells. Following a similar approach to autologous AChs implantation [31], AChs are isolated from an autologous biopsy taken from a lesser-weight-bearing region. However, due to the low cellularity of cartilage tissue, this yields a limited number of cells. To overcome this limitation, chondrocytes can be expanded in vitro through monolayer culture to obtain a sufficient number of cells for incorporation into the TE. Nevertheless, during 2D expansion, chondrocytes tend to lose their chondrogenic phenotype and undergo dedifferentiation, characterized by decreased production of collagen type II and aggrecan, alongside increased expression of fibrocartilage-associated collagen type I [19]. Therefore, it is crucial to understand the cellular mechanisms involved in ACh dedifferentiation and to identify strategies to suppress them. Narcisi et al. and Schofield et al. demonstrated that inhibiting TGFβ and tropomyosin, or CDC42, respectively, during AC expansion promotes chondrogenic redifferentiation [223, 269]. Opting for a less invasive approach that also reduces the risk of additional joint injuries, autologous nasal chondrocytes isolated from nasal septum biopsy have recently emerged as a promising alternative. A study comparing nasal chondrocytes and AChs demonstrated that nasal chondrocytes proliferate faster, exhibit higher chondrogenic capacity, and show less donor age-related dependency [139, 288]. Moreover, nasal chondrocytes have been shown to positively respond to mechanical loading by increasing collagen and GAG synthesis [38]. In addition, a tissue-engineered construct consisting of nasal chondrocytes seeded on a collagen-based scaffold (ChondroGide), known as N-TEC, has been developed and tested in phase 1 (Nose to Knee I, ClinicalTrials.gov Identifier: NCT01605201) and phase 2 (Nose to Knee II, ClinicalTrials.gov Identifier: NCT02673905) clinical trials, demonstrating its potential for repairing AC defects in the knee [79, 221].

As an alternative to fully differentiated chondrogenic cells, primary MSCs have long been considered an attractive cell source for TE. These primary cells represent a heterogeneous population found within the stromal compartment of various tissues and organs, primarily bone marrow, adipose tissue, and synovium. MSCs possess self-renewal capacity and the ability to differentiate into multiple musculoskeletal lineages, including chondrocytes, osteoblasts, and adipocytes [29]. However, studies have demonstrated that MSCs tend to differentiate into a hypertrophic chondrocyte phenotype both in pellets [218] and scaffold-based constructs (e.g., GelMA) when compared to ACh [166, 305]. Hence, further research is essential to prevent this undesired differentiation, with mechanobiological approaches presenting promising strategies to guide MSCs toward a more stable chondrogenic phenotype [218]. To overcome MSC-associated hypertrophic differentiation, chondroprogenitor cells residing in the superficial zone of adult articular cartilage have been identified [17, 68]. Termed articular cartilage-derived progenitor cells (ACPCs), these cells can be clonally expanded in vitro while preserving their chondrogenic potential over extended population doublings and are less susceptible to hypertrophic differentiation than MSCs [166, 205]. Furthermore, ACPCs exhibit enhanced chondrogenesis in response to mechanical stimulation [175, 177, 227].

The following summary captures current understanding and future priorities in cartilage tissue engineering constructs:

- Stiffer hydrogels favour chondrogenic performance in chondrocytic cells (i.e., chondroprogenitor cells (ATDC5) and either bovine or human AChs) [167, 168, 175, 177, 333].
- Both hydrostatic pressure and dynamic compression enhance porcine BMSC chondrogenesis more in stiffer than in softer agarose hydrogels, emphasizing the importance of stiffness–loading interactions [283, 284].
- Faster-relaxing, viscous hydrogels support chondrogenesis in MSCs [123, 124, 311].
- MSC chondrogenesis is improved in oxidized alginate–gelatin matrices with lower porosity [13].
- A stable chondrogenic phenotype is promoted by nano-pillars on soft substrates, whereas nano-holes, nano-gratings, or nano-pillars on stiffer substrates reduce stability [328, 329]
- Given the limitations of AChs [19] and MSCs [218, 305], NChs [139, 288] and ACPCs [166, 205] represent promising cell sources for future mechano-chondrogenic studies.

## Chondrogenic mechanostimuli

Using the TE constructs mentioned above as in vitro models, investigators in the field of in vitro regenerative rehabilitation have explored the impact of mechanical stimuli (Fig. 4B) —such as compression, shear stress, hydrostatic pressure, and tensile strain—on MSC chondrogenesis and the expression of extracellular matrix proteins by chondrogenic cells.

This review highlights compression and shear, the predominant forces experienced by knee articular cartilage, as reported by Chan et al. [44]. Several key factors modulate the effects of mechanical stimuli, including the type of force applied, the waveform characteristics (Fig. 4C) (such as continuous versus intermittent or static versus dynamic), as well as the duration, magnitude, frequency of the forces and the number of axes. In addition to these mechanical parameters as well as the bioreactor apparatus (Fig. 4D), biological factors, such as cell source, cell seeding density, PCM, scaffold, TGFβ-media supplementation and preloading conditions, also significantly influence the cellular response.

### Contact compression

Contact compression applied along the vertical plane results in deformation of the matrix and cells, which also induces, as a side effect, fluid flow (i.e., fluid being drawn in and out of the construct) and nutrient transfer. In compression stimulation, two main configurations are commonly employed: confined and unconfined. In the confined setup, the tissue engineering construct is enclosed within a rigid chamber, preventing outward deformation during compression. This configuration typically requires a rigid porous plate to permit access to media and nutrient supply. In contrast, the unconfined configuration allows lateral expansion of the construct, with compression applied, for instance, between two planar surfaces. As shown in the summary table below (Table 1 and 2), direct compression—particularly uniaxial unconfined compression—is by far the most commonly employed mechanical stimulation in TE.

**Table 1:** Summary table of the literature cited in the text describing the effect of mechanical stimuli on MSC chondrogenesis

Type of Strain	Cell source	Loading Regimen	Bioreactor	Scaffold	Loading Culture media	Preloading duration	Experiment duration	Load effect	Ref
Compression	hBMSCs	**Waveform:** Continuous dynamic **Cycle duration:** 4h per day **Loading duration:** first 7 days **Magnitude:** 7994 Pa **Frequency:** 0.33 Hz	Uniaxial Unconfined	hyaluronan–gelatin-composite	DMEM-HG, ITS + Premix, 37.5 μg/mL AA2P, Dex (10^–7^ M), and **10 ng/mL TGFβ-1**	-	1, 7, 14 and 21 days	14 and 21 days: COL2A1 ↑ ACAN ↑ 21 days: GAG ↑ Collagen ↑	[11]
	rBMSCs	**Waveform:** Continuous dynamic **Cycle duration:** 4h per day **Loading duration:** 3, 7 and 14 consecutive days **Magnitude:** 10% strain **Frequency:** 1 Hz	Uniaxial Unconfined	2% Agarose	DMEM-HG, 1% ITS, 1.25 mg/mL BA, 5.33 µg/mL LA, 40 µg/ml P, 50 µg/mL AA, and 10^–7^ M Dex, and with/without **10 ng/mL TGFβ-1**	20—24 h without **TGFβ**	3, 7 and 14 days	• Without **TGFβ-1:** 3, 7 and 14 days: Col2 ↑ Acan ↑ Tgfβ-1 ↑ With **TGFβ-1:** 14 days: Col2 ↑	[121]
	rBMSCs	**Waveform:** Continuous dynamic **Cycle duration:** 2 or 4 h per day **Loading duration:** 1 and 2 consecutive days **Magnitude:** 15% strain **Frequency:** 1 Hz	Uniaxial Unconfined	2% Agarose	UltraCULTURE, 1% antibiotics, and 0.29 mg/mL L-GLN	44—48 h without **TGFβ**	1 and 2 days	• 2 h loading**:** Col2 ↑ Sox9 ↑ Tgfβ-1 ↑ TgfβRI and II ↑ • 4 h loading**:** 2nd day: Col2 ↑ Sox9 ↑ Tgfβ-1 ↑ TgfβRI and II ↑	[122]
	bBMSCs	**Waveform:** Continuous dynamic **Cycle duration:** 3h per day **Loading duration:** 5 days **Magnitude:** 10% strain **Frequency:** 1 Hz	Uniaxial Unconfined	2% Agarose	DMEM-HG, 1 × PSF, 50µg/mL AA2P, 40µg/mL L-P, 100 µg/mL SP,1 × ITS + Premix, and 0.1µM Dex	3 days without **TGFβ and Dex**	0, 7, 14 and 28 days	14 and 28 days: GAG ↑	[201]
	bBMSCs	**Waveform:** Continuous dynamic **Cycle duration:** 1 or 3 or 20h per day **Loading duration:** 1 day **Magnitude:** 10% strain **Frequency:** 1 Hz	Uniaxial Unconfined	3% Agarose	DMEM-HG, antibiotic/antimycotic, NEAA, 1% ITS + Premix, 50 μg/mL ascorbate, 0.4 mM P, and with/without **100 nM Dex** and **10 ng/ml TGFβ-1**	8 or 16 days without/with **TGFβ** or with **TGFβ and Dex** (respectively)	0, 7, 14 and 28 days	• 8 days preculture**:** No differences between FS vs DC • 16 days preculture**:** Without **TGFβ-1**: pSmad2/3 ↑ With **TGFβ-1**: COL2A1 ↑ COL1A1 ↑ Protein ↑ With **TGFβ-1 + Dex**: COL2A1 ↑ ACAN ↑ COL1A1 ↑ GAG and Protein ↑	[216]
	gBMSCs	**Waveform:** Continuous dynamic **Duration:** without TGF-β1 (1 h or 2 h for 1 day) and with TGF-β1 (2.5h per day for 6 and 14 days and 4 h per day for 6, 14 and 21 days); **Loading duration:** 1-, 2-, and 3-weeks **Magnitude:** 10% strain **Frequency:** 1 Hz	Uniaxial Unconfined	poly(ethylene glycol)-diacrylate (PEGDA) hydrogel	DMEM, 100 nM Dex, 40μg/mL P, 50μg/mL AA, 1% SP, penicillin/streptomycin, insulin-transferrin-selenium acid premix, and with/without **10 ng/mL** **TGFβ-1**	-	1, 6, 14 and 21 days	• Without **TGFβ-1:** SOX9 ↑ ACAN ↑ • With **TGFβ-1** and **2.5 h of loading:** COL2A1 ↑ ACAN ↑ SOX9 ↑ GAG ↑	[290]
	pBMSCs	**Waveform:** Continuous Intermittent dynamic **Cycle duration:** 1h per day **Loading duration:** 5 days per week **Magnitude:** 10% strain **Frequency:** 0.5 Hz	Uniaxial Unconfined	2% Agarose	DMEM GlutaMAX, penicillin/streptomycin, 100 µg/mL SP, 40 µg/mL L-P, 50 µg/ml L-AA2P, 1 mg/mL BSA, 1 × ITS, 100 nM Dex, and **10 ng/mL TGFβ-3**	4 days without **TGFβ and Dex**	0, 14 and 42 days	42 days: GAG ↓ Collagen ↓	[293]
	eBMSCs	**Waveform:** Intermittent dynamic **Cycle duration:** 45 min on/45 min off per day; **Loading duration:** 21 days **Magnitude:** 2.5% strain (static 7.5% offset) **Frequency:** 0.3 Hz	Uniaxial Unconfined	2% Agarose	DMEM-HG, 1% ITS, 0.1 μM Dex, 37.5 μg/mL AA2P, and with/without **10 ng/mL TGFβ-1**	-	21 days	• Without **TGFβ-1:** GAG ↑ Collagen → • With **TGFβ-1:** GAG ↓ Collagen ↓	[149]
	pBMSCs	**Waveform:** Continuous dynamic **Cycle duration:** 1h per day **Loading duration:** 5 days per week **Magnitude:** 10% strain **Frequency:** 1 Hz	Uniaxial Unconfined	2% Agarose	DMEM-HG, penicillin/streptomycin, 100 µg/ml SP, 40 µg/mL L-P, 1.5 mg/mL BSA, 4.7 µg/mL LA, 1 × ITS, 50 µg/mL L-AA2P, 100 nM Dex, and with/without **10 ng/mL TGF-β3**	3 days with **TGF-β**	0, 21 and 42 days	• Without static preculture independent of TGF-β supplementation: GAG ↓ • With static preculture independent of TGF-β supplementation: GAG → Collagen →	[294]
	bBMSCs	**Waveform:** Continuous dynamic **Cycle duration:** 1 or 4 h per day **Loading duration:** 3 weeks **Magnitude:** 10% strain **Frequency:** 0.1 or 1 Hz	Uniaxial Unconfined	4% Agarose	DMEM-HG, 1X PSF, 0.1 μM Dex, 50 μg/mL AA2P, 40 μg/mL L-P, 100 μg/mL SP, ITS, 1.25 mg/ml BSA, 5.35 μg/ml LA, and with/without **10 ng/mL TGFβ-3**	3 days with or without **TGFβ** or 3 weeks with **TGFβ**	3 weeks	• Without static preculture**:** Without **TGFβ-3 after 3 weeks:** GAG ↓ Collagen ↓ COL2A1 → ACAN → With **TGFβ-3 after 3 weeks:** GAG ↓ Collagen ↓ COL2A1 → ACAN ↑ • With static preculture**:** No differences between FS vs DC	[120]
	hBMSCs	**Waveform:** Intermittent dynamic **Cycle duration:** 1h per day **Loading time points:** 0,7, 14- and 21-days **Magnitude:** 10% strain **Frequency:** 1 Hz	Uniaxial Unconfined	2% Agarose	DMEM-HG, 100 U/mL penicillin/streptomycin, 100 mg/mL SP, 40 mg/mL L-P, 1.5 mg/mL BSA, 4.7 mg/mL LA, 1 × ITS, 50 mg/mL L-AA2P, 100 nM Dex, and **10 ng/mL TGFβ-3**	60h with **TGF-β**	21 days	21 days: COL2A1 ↑ ACAN ↑ COL1A1 ↓ (annular)	[110]
	pBMSCs	**Waveform:** Continuous dynamic **Cycle duration:** 3h per day **Loading duration:** 7 days per week over 21- or 42-days** Magnitude:** 10% strain (static 1% offset) **Frequency:** 1 Hz	Uniaxial Unconfined	Fibrin hydrogel	DMEM-HG, penicillin/streptomycin, 100 KIU/mL aprotinin, 0.25 μg/mL amphotericin B, 100 μg/mL SP, 40 μg/mL L-P, 1.5 mg/mL BSA, 4.7 μg/mL LA, 1 × ITS, 50 μg/mL L-AA2P, 100 nM Dex, and **10 ng/mL TGF-β3**	0 or 21 days with **TGF-β**	21 or 42 days	• Without static preculture: 21 days: COL2A1 ↑ COL1A1 ↓ ACAN → SOX9 → COL10A1 → GAG ↓ Collagen ↓ 21—42 days: GAG ↑ • With static preculture: 42 days: GAG ↑ Collagen →	[292]
	hBMSCs	**Waveform:** Continuous dynamic **Cycle duration:** 2h per day **Loading time points:** 7 days per week for 3 weeks **Magnitude:** 5% strain **Frequency:** 1 Hz	Uniaxial Unconfined	poly L-lactide-co-caprolactone (PLCL)/chitosan scaffolds	DMEM-HG, 10^–7^ M Dex, 1% ITS + premix, 50 mg/mL AA, 1 mM SP, 4 mM P, and **10 ng/mL TGF-β3**	3 weeks with **TGF-β**	3 weeks	3rd week: COL2A1 ↑ ACAN ↑ COL10A1 ↓ RUNX2 ↓ GAG ↑ COL II ↑	[345, 346]
	hBMSCs	**Waveform:** Continuous dynamic **Cycle duration:** 1, 3, 6 h **Loading time points:** 1 day or 2 weeks **Magnitude:** 5, 10 or 50% strain **Frequency:** 1 Hz	Uniaxial Unconfined	Silk-fibroin or Silk fibroin-gelatin/chondroitin sulfate/hyaluronate scaffolds	DMEM-HG, 2 mM GlutaMAX™, 10 mM HEPES, 0.1 mM NEAA,0.5 μg/mL amphotericin B, 50 U/mL penicillin G sodium, 50 μg/mL streptomycin, 0.4 mM L‐P, 0.1 mM AA, 1.25 mg/mL BSA, 0.1 μM Dex, 1% ITS + 1, and **10 ng/mL TGF‐β3**	1 week with **TGF-β**	1 day or 2 weeks	2nd week: GAG ↑ Aggrecan ↑	[266]
	hSMSCs	**Waveform:** Continuous dynamic **Cycle duration:** 1 h per day **Loading time points:** 28- or 7-days **Magnitude:** 10 kPa **Frequency:** 0.25 Hz	Uniaxial Unconfined	2% Agarose	DMEM-HG containing 10^–7^ M Dex, 50 μg/mL AA phosphate, 100 μg/mL SP, 40 μg/mL P, 1% ITS, and **10 ng/ml TGF-β3**	0 or 21 days with **TGF-β**	28 or 7 days	• Without static preculture**:** SOX9 ↓ COL2A1 ↓ ACAN ↓ RUNX2↓ COL1A1 ↓ COL10A1 ↓ • With static preculture**:** SOX9 ↑ COL2A1 ↑ ACAN↑ RUNX2↓ COL1A1 ↓ COL10A1 ↓	[87]
	**raBMSCs**	**Waveform:** Intermittent static **Cycle duration:** (20 min on/ 20 min off) × 8 times per day **Loading time points:** 7- or 14- or 28-days **Magnitude:** 0.5% strain	Uniaxial Confined	hyaluronic acid methacryloyl (HAMA) and sodium alginate (Alg)	ITS, Dex and **TGF-β3**	-	7- or 14- or 28-days	• 28 days**:** COL X↓	[49]
Compression and Shear	hBMSCs	**Waveform:** Continuous dynamic **Cycle duration:** 1 h per day **Loading duration:** 14 days **Comp. Magnitude:** 10—20% strain **Comp. Frequency:** 1 Hz **Shear:** 25° at 1Hz	Multiaxial Unconfined	Fibrin-polyurethane scaffold	DMEM-HG, ITS, Penicillin/Streptomycin, 1% NEAA, 50 μg/mL AA2P, 5 μM 5 EACA, 10^−7^ M Dex, and **0 ng/mL or 1 ng/mL or 10 ng/mL TGF-β1**	7 days without/with **TGF-β**	14 days	• TGF-β1 supplemented to the media: 0 ng/mL: Overall GAG ↑ COL2A1 ↑ ACAN↑ COL10A1↑ TGFβ-1 and -3 ↑	[172, 179, 180]
	hBMSCs	**Waveform:** Continuous dynamic **Cycle duration:** 1 h per day **Loading duration:** 14 days **Magnitude:** 5, 10, 20% strain (static 10% offset) **Frequency:** 0.1 or 1 Hz **Shear:** 25° at 0.1 or 1Hz	Multiaxial Unconfined	Fibrin-polyurethane scaffold	DMEM-HG, ITS, Penicillin/Streptomycin, 1% NEAA, 50 μg/mL AA2P, 5 μM EACA, 10^−7^ M Dex, and **1 ng/ml TGF-β1**	7 days with **TGF-β**	14 days	• 1 Hz and 20% Dynamic compression Overall GAG ↑ COL2A1 ↑ ACAN↑ COL1A1↑ COL10A1↑ TGFβ-1 and -3 ↑	[172, 179, 180]
	hBMSCs	**Waveform:** Continuous dynamic **Cycle duration:** 1 h per day **Loading duration:** 5 consecutive days over 3 weeks **Comp. Magnitude:** 10—20% strain **Comp. Frequency:** 1 Hz **Shear:** 25° at 1Hz	Multiaxial Unconfined	Fibrin-polyurethane scaffold	DMEM-HG, 2.2 g/L NaHCO3, NEAA, 11.5 mg/L L-P, 50 μg/mL AA2P, ITS + 1, 5 ng/mL sodium selenite, 0.5 mg/mL BSA and 4.7 μg/mL LA, penicillin/streptomycin, 5μM EACA	2—4 days without** TGF-β**	3 weeks	• Both Dynamic compression and shear: Overall GAG ↑ COL2A1 ↑ ACAN↑ SOX9 ↑	[268]
	hBMSCs	**Waveform:** Continuous dynamic **Cycle duration:** 1 h per day **Loading duration:** 5 consecutive days over 3 weeks **Comp. Magnitude:** 10% strain (static 10% offset) **Comp. Frequency:** 1 Hz **Shear:** 25° at 1Hz	Multiaxial Unconfined	Fibrin-polyurethane scaffold	DMEM-HG, 0.11 g/L SP, 50 μg/mL L-P, 10^−7^ M dexamethasone, 1% ITS, 1% NEAA, 0.1% Primocin, and 5 μM 6‐aminocaproic acid	-	4 weeks	• Both Uniform and Asymmetric seeding: ACAN↑ SOX9 ↑ COL10A1 ↑	[82, 83]
	hBMSCs	**Waveform:** Continuous dynamic **Cycle duration:** 1 h per day **Loading duration:** 21 consecutive days **Comp. Magnitude:** 10—20% strain **Comp. Frequency:** 1 Hz **Shear:** 25° at 1Hz	Multiaxial Unconfined	methylcellulose-polyurethane scaffold	DMEM-HG, 2.2 g/L NaHCO_3_, NEAA, 11.5 mg/L L-P, 50 μg/mL AA2P, ITS + 1, 5 ng/mL sodium selenite, 0.5 mg/mL BSA 4.7 μg/mL LA, penicillin/streptomycin, and 10^−7^ M Dex	2 days without** TGF-β**	3 weeks	3rd week: COL2A1 ↑ ACAN ↑ SOX9↑ COL10A1 ↑ COL2A1/COL10 ↑ GAG ↑	[56]
	hBMSCs	**Waveform:** Continuous dynamic **Cycle duration:** 1 h per day **Loading duration:** 5 consecutive days over 25 days **Comp. Magnitude:** 10—30% strain **Comp. Frequency:** 1 Hz **Shear:** 1Hz	Multiaxial Unconfined	Fibrin-polyurethane	DMEM-HG, 0.11 g/L SP, 50 μg/mL L-AA2P, 100 nM Dex, Corning ITS + Premix, 1% NEAA,1% (v/v) P/S, and 5 μM 6-aminocaproic acid	-	25 days	14 days: SOX9↑ SOX9/RUNX2↑ 25 days: ACAN ↑ COL10A1 ↑ COL1A1 ↑ GAG retained ↑ GAG secreted ↓	[158]

hBMSCs: human Bone Marrow stromal cells; raBMSCs: rat Bone Marrow stromal Cells rBMSCs: rabbit Bone Marrow stromal Cells; bBMSCs: bovine Bone Marrow stromal cells; gBMSCs: goat Bone Marrow stromal cells; pBMSC: porcine Bone marrow stromal cells; eBMSCs: equine Bone marrow stromal cells; hSMSCs: human synovium-derived mesenchymal stem cells; DMEM-HG: DMEM-HG: Dulbecco’s Minimal Essential Medium with 4.5 g/L glucose (high glucose); ITS: Insulin-Transferrin-Selenium; AA: ascorbic acid; AA2P: ascorbate 2-phosphate; Dex: ascorbate 2-phosphate; TGFβ: transforming growth factor; BA: bovine albumin; LA: linoleic acid; P: proline; L-GLN: L-glutamine; PSF: penicillin/streptomycin/ fungizone; SP: Sodium pyruvate; FS: Free-swelling control; DC: Dynamic compression group; BSA: Bovine serum albumin; EACA: $\varepsilon$-amino-caproic acid; HEPES: N-(2-Hydroxyethyl)piperazine-N'-(2-ethanesulfonic acid)

Note: Unless otherwise specified, the load effect results presented in the table are reported relative to the free-swelling control

**Table 2:** Summary table of the literature cited in the text describing the effect of mechanical stimuli on chondrogenic phenotype and hyaline cartilage-matrix production of chondrocytes

Type of Strain	Cell source	Loading Regimen	Bioreactor	Scaffold	Culture media	Preloading duration	Experiment duration	Load effect	Ref
Compression	bAChs	**Waveform:** Continuous dynamic **Cycle duration:** 48 h **Loading duration:** 48 h **Comp. Magnitude:** 15% strain **Comp. Frequency:** 0.3, 1 and 3 Hz	Uniaxial Unconfined	3% Agarose	DMEM, 20% FCS, 0.5 mM L-P and, and 1 µCi/mL proline	16 h without **TGF-β**	48 h	1 Hz dynamic compression: GAG↑ Static and 0.3 Hz dynamic compression: GAG↓	[161]
	bAChs	**Waveform:** Intermittent dynamic **Cycle duration:** (3 × 1h on/1h off) per day **Loading duration:** 5 days per week **Comp. Magnitude:** 10% strain **Comp. Frequency:** 1 Hz	Uniaxial Unconfined	2% agarose	DMEM and 50 g/mL AA	-	0, 3, 7, 14 and 21 days	Day 21: GAG↑	[202]
	bAChs	**Waveform:** Continuous dynamic or static **Cycle duration:** 24h **Loading duration:** 24h **Comp. Magnitude:** static (10, 30 or 50% strain) or dynamic: 5% strain (10 or 50% offset) **Comp. Frequency:** 0.001 or 0.1 Hz	Uniaxial Unconfined	ply-glycolic acid (PGA) scaffold	DMEM with 4.5g/L glucose. gentamicin, SP, glutamine, NEAA, calf thymus DNA, L-P, papain, sodium phosphate, cysteine-HCI, bovine chondroitin sulphate, FBS, and 50 μg/mL ascorbate	3 weeks without **TGF-β**	24h	50% Dynamic compression at 0.1 Hz: GAG↑ compared to both free swelling and static compression 10% Dynamic compression at 0.1 Hz: GAG↑	[60]
	bAChs	**Waveform:** Continuous or Intermittent dynamic **Cycle duration:** Continuous** (**1.5, 3, 6, 12, 24 and 48 h) or Intermittent ( 1.5h on/ 1.5h off, 3h on/ 3h off, 6h on/6h off, 12h on/ 12h off for a total period of 48 h) **Loading duration:** 48 h **Comp. Magnitude:** 15% strain **Comp. Frequency:** 1 Hz	Uniaxial Unconfined	3% Agarose	DMEM, 20% FCS,1 µCi/mL thymidine, and 10 μCi mL^−135^SO_4_	24 h without **TGF-β**	48 h	12h Intermittent loading: GAG↑	[51, 52]
	bAChs	**Waveform:** Continuous dynamic or static **Cycle duration:** 24 h per day **Loading duration:** 0-, 10- and 20-days **Comp. Magnitude:** Static: 10% strain or Dynamic: 4% strain (static 10% offset) **Comp. Frequency:** 0.1 and 1 Hz	Uniaxial Unconfined	Fibrin gel	DMEM, 10% FBS, 0.1 mM NEAA, 4 mM L-glutamine, 1 mM SP, 5 μg/mL gentamicin sulfate, 2 mg/mL aminocaproic acid, and 50 μg/mL AA	3 days without **TGF-β**	0, 10 and 20 days	0.1 Hz Dynamic compression: GAG↑ compared to static compression Compression: GAG↓ compared to free swelling	[125]
	bAChs	**Waveform:** Intermittent dynamic **Cycle duration:** 6 min every 48h **(**400cycles) or 30 min every 48h (2000 cycles) three times per week **Loading duration:** 2- or 4-weeks **Comp. Magnitude:** 5, 10 or 20% strain **Comp. Frequency:** 1 Hz	Uniaxial Unconfined	Calcium phosphate substrate	Ham’s F12, 25 mM HEPES and 5 or 20% FBS, 100 μg/mL AA, and penicillin/streptomycin/ amphotericin	4 weeks without **TGF-β**	1, 2 or 4 weeks	4 weeks stimulated: GAG↑ Collagen↑	[310]
	bAChs	**Waveform:** Continuous dynamic **Cycle duration:** 3 h per day **Loading duration:** 5 days per week **Comp. Magnitude:** 10% strain (static 10% offset) **Comp. Frequency:** 1 Hz	Uniaxial Unconfined	2% agarose	DMEM-HG, 1 × PSF, 0.1 μM Dex, 50 μg/mL AA2P, 40 μg/mL L-P, 100 μg/mL SP, and 1 × ITS with a discontinuous TGF-β strategy (consisting of 14 days with **10 ng/mL TGF-β3** followed by either 28 or 42 days without supplementation of **TGF-β3**)	0 or 14 days with **TGF-β**	56 or 28 days	Immediate dynamic compression: GAG↓ Collagen ↓ Delayed dynamic compression: GAG → Collagen →	[184]
	rAChs	**Waveform:** Continuous or Intermittent dynamic **Cycle duration:** Continuous: 24h or Intermittent: 6h on/6h off or 12 h on/12 h off or 24 h on **Loading duration:** 3-days **Comp. Magnitude:** 10% strain **Comp. Frequency:** 0.01, 0.05, 0.1 or 0.5 Hz	Uniaxial Unconfined	poly(L-lactide-co-epsilon-caprolactone) (PLCL) sponge	DMEM, 10% FBS, 10 mM HEPES buffer, 44 mM bicarbonate of soda, and penicillin/ streptomycin	3 days without **TGF-β**	3 days	24 h continuous dynamic compression: Col2 ↑ Col1 ↑ Acan ↑ compared to intermittent dynamic compression	[330]
	roAChs	**Waveform:** Continuous dynamic **Cycle duration:** 24h **Loading duration:** 24h **Comp. Magnitude:** 5%, 10%, or 15% **Comp. Frequency:** 0.5 Hz, 1.0 Hz, 2.0 Hz, or 3.0 Hz	Uniaxial Unconfined	3% agarose	DMEM, 10% FBS and 50 µg/mL AA	-	1, 3 or 7 days	10% DC and 1 Hz: GAG ↑	[297]
	bAChs	**Waveform:** Intermittent or Continuous dynamic **Cycle duration:** Intermittent loading: (3 × 1h on/1h off) per day or Continuous loading: 3h or 6h per day **Loading duration:** 5 days per week **Comp. Magnitude:** 10% strain **Comp. Frequency:** 1 Hz	Uniaxial Unconfined	2% agarose	DMEM	2 days without **TGF-β**	28 days	3 or 6 h Continuous Dynamic compression: Collagen type II ↑ Collagen type IX ↑ compared to intermittent loading and free swelling	[229]
	bAChs	**Waveform:** Intermittent or Continuous dynamic **Cycle duration:** Intermittent loading: (6 × 1h on/1h off) per day or Continuous loading: 24h per day **Loading duration:** 7 days **Comp. Magnitude:** 20% strain **Comp. Frequency:** 0.3 Hz	Uniaxial Unconfined	poly(ethylene glycol) (PEG) hydrogel	DMEM, 10% FBS, 0.04 mM L-P, 50 mg/L L-AA, 10 mM HEPES buffer, 0.1 M MEM-NEAA, 1% penicillin/streptomycin, 0.5 mg/mL fungizone, and 20 mg/mL gentamicin	24 h without **TGF-β**	7 days	Intermittent Dynamic compression: COL2A1 ↑ MMP ↑ GAG ↓ compared to continuous loading	[231]
	cAChs	**Waveform:** Continuous dynamic **Cycle duration:** 3 h per day **Loading duration:** 5 days per week **Comp. Magnitude:** 5% strain (static 10% offset) **Comp. Frequency:** 1 Hz	Uniaxial Unconfined	2% Agarose	DMEM, 1% ITS + Premix, 50 μg/mL L-P, 0.1 μM Dex, 0.9 mM SP, antibiotics, 50 μg/mL ascorbate, and **10 ng/mL TGF-β3**	0, 2 or 4 weeks with **TGF-β**	4, 6 or 8 weeks	GAG → Collagen →	[20]
	bAChs	**Waveform:** Continuous dynamic **Cycle duration:** 24 h **Loading duration:** 24h **Comp. Magnitude:** 2.5% strain (static 7% offset) **Comp. Frequency:** 1 Hz	Uniaxial Unconfined	2% Agarose	DMEM-HG, 0.1 mM NEAA, 0.4 mM P, 100 U/mL PSA (penicillin, streptomycin, amphotericin), 10 μg/mL ascorbate, and 1% ITS	18h without **TGF-β**	24h	GAG ↑	[41]
	hAChs	**Waveform:** Continuous dynamic **Loading duration:** 14 days **Comp. Magnitude:** 10% **Comp. Frequency:** 0.3 Hz	Uniaxial Unconfined	Collagen type I hydrogel	DMEM medium and 2 M HEPES	overnight without **TGF-β**	14 days	COL2A1 → COL1A1 → ACAN →	[225]
	hAChs	**Waveform:** Continuous dynamic **Loading duration:** 28 days **Comp. Magnitude:** 10% **Comp. Frequency:** 0.3 Hz	Uniaxial Unconfined	Collagen type I hydrogel	DMEM medium and 2 M HEPES	overnight without **TGF-β**	28 days	COL2A1 ↑ COL1A1 ↑ ACAN →	[224]
	hAChs	**Waveform:** Continuous dynamic **Cycle duration:** 1, 3, 6 h **Loading time points:** 1 day or 2 weeks **Magnitude:** 5, 10 or 50% strain **Frequency:** 1 Hz	Uniaxial Unconfined	Silk-fibroin or Silk fibroin-gelatin/chondroitin sulfate/hyaluronate scaffolds	DMEM-HG, 2 mM GlutaMAX™, 10 mM HEPES, 0.1 mM NEAA,0.5 μg/mL amphotericin B, 50 U/mL penicillin G sodium, 50 μg/mL streptomycin, 0.4 mM L‐P, 0.1 mM AA, 1.25 mg/mL BSA, 0.1 μM Dex, 1% ITS + 1, and **10 ng/mL TGF‐β3**	1 week with **TGF-β**	1 day or 2 weeks	COLII ↑ Aggrecan ↑ GAG ↑	[266]
	hAChs	**Waveform:** Continuous dynamic **Cycle duration:** 1h per day **Loading duration:** 14 days **Comp. Magnitude:** 5% strain **Comp. Frequency:** 0.2 Hz	Uniaxial Unconfined	poly-D,L-lactic acid/polyethylene glycol/poly-D,L-lactic acid (PEG-PDLLA)/ polycaprolactone–poly(ethylene glycol)-polycaprolactone (PEG-PCL) hydrogel	DMEM-HG, 1% antibiotics–antimycotics, 0.1 μM Dex, 50 μg mL^−1^ AA2P, 40 μg mL^−1^ L-P, 10 μg mL^−1^ ITS, and **10 ng mL**^**−1**^** TGF-β3**	2 weeks with **TGF-β**	2 weeks	COL2A1 ↑ ACAN ↑ SOX9 ↑	[331]
	ATDC5 cells	**Waveform:** Continuous dynamic or static **Cycle duration:** 2h every two days **Loading duration:** 21 days **Comp. Magnitude:** 20% strain **Comp. Frequency:** 0.0017 Hz	Uniaxial Confined	gelatin methacryloyl (GelMA) hydrogel	DMEM/F-12 medium, 10% FBS, and 1% antibiotics	3 days without **TGF-β**	21 days	Col2a1 ↑ Acan ↑ Sox9 ↑ Prg4 ↑ (only day 21) compared to static compression and free-swelling	[333]
Shear	bAChs	**Waveform:** Continuous fluid shear **Loading duration:** 7- or 15-days **Fluid shear rate:** 1 μm/s **Pressure:** -	Uniaxial (perfusion)	poly-L-lactic acid (PLLA)/ polyglycolic acid (PGA) disks	Ham’s F-12, 10% FBS, penicillin/ streptomycin, and 25 µg/mL L-AA	24 h	2 weeks	GAG ↑ Collagen ↑	[245]
	bAChs	**Waveform:** Continuous fluid shear **Cycle duration:** 24h **Loading duration:** 24h **Shear rpm:** 80,120 or 160 rpm	Multiaxial (spinner flask)	polyglycolic acid (PGA)	DMEM, 10% FBS, 0.584 mg/mL glutamine, penicillin/ streptomycin, 10mM HEPES, 0.1 mM NEAAA, 0.4 mM P, and 50 mg/mL AA	3 days	1 day	GAG ↑	[91]
	bAChs	**Waveform:** Continuous fluid shear **Loading duration:** 7- or 15-days **Fluid shear rate** 0.33 mL/min **Pressure**: -	Uniaxial (perfusion)	Collagen sponge	Ham’s F12, 10% FBS, and penicillin/ streptomycin	3 days	7 or 15 days	GAG ↓ COL2A1 ↓ ACAN ↓	[213]
	hAChs	**Waveform:** Intermittent dynamic contact shear **Cycle duration:** 400 or 2000 cycles per day followed by 48h off **Loading duration:** 1 or 4 weeks (three times per week) **Shear. Magnitude:** 2, 6, 12% strain **Shear. Frequency:** 1 Hz	Uniaxial (contact shear)	Porous calcium polyphosphate substrate	Ham's F12, 25 mM HEPES, and 5% FBS	4 weeks	1 or 4 weeks	One week: Collagen ↑ GAG ↑ Four weeks: Collagen ↑	[308, 309]
	bAChs	**Waveform:** Intermittent dynamic **Loading duration:** 400 cycles followed by 24h off **Shear:** 2% strain at 1 Hz	Uniaxial (contact shear)	Calcium phosphate ceramic surface	Culture media, penicillin/streptomycin, and 0.25 µg/mL amphotericin B	4 weeks	4 weeks	GAG ↑	[308, 309]
	hAChs	**Waveform:** Continuous fluid shear **Loading duration:** 2 weeks **Fluid shear rate:** 0.5 mL/min **Pressure:** 4.6, 14, 25 and 56 mPa	Uniaxial (perfusion)	polyestherurethane foam (DegraPol®)	DMEM, SP, Hepes buffer, antibiotics (penicillin/streptomycin), L-glutamine, L-AA, insulin, and 10% FCS	24 h	2 weeks	Lower shear stress: GAG ↑	[256]
	bAChs	**Waveform:** Continuous fluid shear **Loading duration:** 2 weeks **Fluid shear rate:** 0.2 mL/min **Pressure:** 1.2 mPa or 6.7 mPa	Uniaxial (perfusion)	polyestherurethane foam (DegraPol®)	DMEM, SP, HEPES buffer, antibiotics (penicillin, streptomycin), L-glutamine, insulin, and 10% FCS	24 h	2 weeks	1.2 mPa shear stress: GAG ↑ compared to free-swelling and 6.7 shear stress	[255]
	bAChs	**Waveform:** Continuous contact shear **Loading duration:** 1 h per day over 5 days **Shear:** 25° at 0.01, 0.1 or 1 Hz	Uniaxial (contact shear)	Fibrin-polyurethane scaffold	DMEM, antibiotics, 10%FCS, 50 mg/mL AA, 40 mg/mL L-P, NEAA, and 500 KIU/mL aprotinin	6 days	5 days	28 mm/sec shear stress: PRG4 ↑ COMP ↑ HAS1 ↑	[321]
	bAChs	**Waveform:** Continuous fluid shear **Loading duration:** 1,7- or 14-days **Fluid shear rate:** 3 mL/min **Pressure:** -	Tubular perfusion	2% Alginate	DMEM/F12 media, 50 mg/mL AA2P, 1 mg/mL BSA, 1.2 mg/mL sodium bicarbonate, 0.1% penicillin/streptomycin, 0.1% SP, and 10% FBS	-	1,7 or 14 days	14th day: ACAN ↑ COL2A1 ↑ SZP ↑ COL1A1 ↑	[339]
Compression and shear	bAChs	**Waveform:** Continuous static or dynamic **Cycle duration:** 1 h per day **Loading duration:** 37 days **Comp. Magnitude:** static (10% strain) or dynamic (7% strain) **Comp. Frequency:** 0.3 Hz **Fluid shear rate:** 1—3mL/min	Uniaxial Unconfined (with perfusion)	Polyglycolic acid (PGA)	DMEM-HG, 10% FBS, 10 mM HEPES, 0.1 mM NEAA, 0.4 mM P, 50 mg/mL L-AA, and penicillin/streptomycin	30 days without** TGF-β**	37 days	Static and dynamic compression: Collagen ↓	[271]
	bAChs	**Waveform:** Continuous dynamic **Cycle duration:** 1 h × 2 per day **Loading duration:** 5 consecutive days **Comp. Magnitude:** 10—20% strain **Comp. Frequency:** 0.1 Hz **Shear:** 25 at 0.1 Hz	Multiaxial Unconfined	Fibrin-polyurethane scaffold	DMEM, antibiotics, 10% FCS, 50 mg/mL of AA, 40 mg/mL of L-P, NEAA, and 500 KIU/mL of aprotinin	5 days without** TGF-β**	5 days	PRG4 ↑ ACAN ↑ COMP↑ COL2A1 ↑ PRG4 released ↑ COMP released ↑ HA released ↑	[94]
	bAChs	**Waveform:** Continuous dynamic **Cycle duration:** 400 cycles per day **Loading duration:** 3 times in 1 week **Comp. Magnitude:** 2% or 5% strain **Comp. Frequency:** 0.5 Hz **Shear:** 2% or 5% strain at 0.5 Hz	Multiaxial Unconfined	Calcium phosphate substrate	Ham’s F12, 25 mM HEPES, 20% FBS, and 100 µg/mL AA	4 weeks without** TGF-β**	1 week	5% compression and 5% shear: GAG ↑ Collagen ↑	[307]
	cAChs	**Waveform:** Continuous dynamic **Cycle duration:** 3 h per day **Loading duration:** 5 days per week **Comp. Magnitude:** 10% strain **Comp. Frequency:** 0.5 Hz **Shear:** 180° (1.25 revolutions per second)	Uniaxial Unconfined	2% Agarose	DMEM, 1% ITS + Premix, 50 μg/mL L-P, 0.1 μM Dex, 0.9 mM SP, antibiotics, 50 μg/mL ascorbate, and **10 ng/mL TGF-β3**	2 weeks with **TGF-β**	6 weeks	No differences	[20]
	hFECs	**Waveform:** Intermittent dynamic **Cycle duration:** 10 min per day **Loading duration:** 5 days per week **Comp. Magnitude:** 8.7% strain **Comp. Frequency:** 0.05 Hz **Shear:** 3 rpm rotational speed	Multiaxial Unconfined	polyglycolic acid (PGA)–alginate constructs	DMEM, 3.7g/L sodium hydrogen carbonate, 10 mM HEPES, 0.4mM L-P, 0.1 mM NEAA,10% (v/v) FBS, and 0.5% antibiotic antimycotic solution	2.5 or 4 weeks (under either shaking or perfusion culture) without** TGF-β**	1 or 1.5 weeks	• Shaking: GAG ↑ Collagen ↑ • Perfusion: 4 weeks perfusion + 1-week multiaxial loading: Collagen ↑ 2.5 weeks perfusion + 2.5 weeks multiaxial loading: GAG ↓	[274]
	hAChs	**Waveform:** Continuous dynamic **Cycle duration:** 1 h per day **Loading duration:** 6 days per week over 3 weeks **Comp. Magnitude:** 10—20% strain **Comp. Frequency:** 1 Hz **Shear:** 25° at 1Hz	Multiaxial Unconfined	Fibrin-polyurethane scaffold	DMEM, antibiotics, 10% FCS, 50 mg/mL AA, 40 mg/mL L-P, NEAA, and 500 KIU/mL aprotinin	1 week without** TGF-β**	3 weeks	Lubricin	[95]
	bAChs	**Waveform:** Continuous dynamic **Cycle duration:** 30 min per day **Loading duration:** 21 days **Comp. Magnitude:** 18% strain **Comp. Frequency:** 8—14 mm/s **Contact shear:** 8—14 mm/s **Perfusion rate:** 7.72 or 14.82 mL/min	Multiaxial Unconfined (perfusion, compression and contact shear)	3% Agarose	DMEM, 10% FBS, 1% antibiotics, and 100mg/mL AA	7 days without** TGF-β**	21 days	GAG →	[249]
	hAChs	**Waveform:** Continuous dynamic **Cycle duration:** 1 h per day **Loading duration:** 7- or 14-days **Comp. Magnitude:** 20% strain **Comp. Frequency:** 0.5 Hz **Fluid shear rate** 0.1 mL/min	Uniaxial Unconfined (with perfusion)	2% Alginate	DMEM, ITS^+1^, 1.25 mg/mL human serum albumin,10^−7^ M Dex, 0.1 mM AA2P, and penicillin/ streptomycin/gentamycin	24—48h without** TGF-β**	7 or 14 days	No statistically significant differences in chondrogenic gene expression GAG ↓ (trend)	[97]
	hAChs	**Waveform:** Continuous dynamic **Cycle duration:** 1 h per day **Loading duration:** 1- or 14-days **Comp. Magnitude:** 10 or 20 or 30% strain **Comp. Frequency:** 1 Hz **Sliding** s**hear:** 0.5 or 1.0 or 1.5 mm	Uni or biaxial Unconfined	gelatin methacryloyl (GelMA) and hyaluronic acid methacrylate (HAMA) hydrogel	DMEM, 2 mM GlutaMAX™, 10 mM HEPES, 0.1 mM NEAA, 50 U/mL penicillin, 50 µg/mL streptomycin, 0.5 µg/mL amphotericin B, 0.4 mM L-P, 0.1 mM L-AA, 100 × dilution ITS-G, 1.25mg/mL BSA, 0.1μM Dex, and **10 ng/mL TGF-β3**	14 days with** TGF-β**	1 or 14 days	Retained GAG → Secreted GAG ↓ COL2A1 ↑ PRG4↑ COL10A1 ↓ COL II ↑ COL I ↓	[207]
	hAChs	**Waveform:** Continuous dynamic **Cycle duration:** 1 h per day **Loading duration:** 7 or 14 days **Loading Magnitude and frequency:** Compression only: (300 mbar applied pressure at 1 Hz or multi-directional mechanical stimulation: 300 mbar positive pressure, − 350 mbar negative pressure, at 0.33 Hz)	Multiaxial Confined (Cartilage-on-chip)	2% Agarose	DMEM, 1 × ITS premix, penicillin/streptomycin, 4 mM P, 50 μg/mL AA2P, 1 × SP, **20 ng ml − 1 TGF-β3**, and 10^−7^ M Dex	1 or 7 days with** TGF-β**	7 or 14 days	• Multi-directional mechanical: 7 days of culture: COL2A1 ↑ COL1A1 ↓ 14 days of culture: GAG ↑ (also showed an increase under the compression-only condition) 7 days free-swelling preculture followed by 7 days of mechanical culture: Aggrecan, COL II and COL VI ↑ (also showed an increase under the compression-only condition)	[236]

**bAChs:** bovine articular chondrocytes; **rAChs:** rabbit articular chondrocytes; **roAChs:** rodent articular chondrocytes; **cAChs**: canine articular chondrocytes; **hAChs:** human articular chondrocytes; **hFECs**: human foetal epiphyseal chondrocytes **DMEM:** DMEM-HG: Dulbecco’s Minimal Essential Medium; **ITS**: Insulin-Transferrin-Selenium**; AA:** ascorbic acid; **AA2P:** ascorbate 2-phosphate; **Dex:** ascorbate 2-phosphate; **TGFβ:** transforming growth factor; **BA**: bovine albumin; **LA:** linoleic acid; **P:** proline; **L-GLN:** L-glutamine; **PSF**: penicillin/streptomycin/ fungizone; **SP**: Sodium pyruvate; **FS:** Free-swelling control; **DC:** Dynamic compression group; **BSA:** Bovine serum albumin; **EACA:** ε-amino-caproic acid; **FBS:** Foetal Bovine Serum; **FCS:** Foetal calf serum; **HEPES:** N-(2-Hydroxyethyl)piperazine-N′-(2-ethanesulfonic acid);

Note: Unless otherwise specified, the load effect results presented in the table are reported relative to the free-swelling control.

## Exogenous TGFβ-free culture media

- BMSCs

Huang et al. [121] and [122], Mauck et al. [290] and Kisiday et al. [149] showed that dynamic compression without exogenous TGFβ-media supplementation and without significant TGFβ-free preculture time, promotes BMSCs chondrogenesis [121, 122, 149, 201]. In contrast, Huang et al. [120] reported opposite findings [120], while Schätti et al. found that dynamic compression alone did not affect hBMSC chondrogenesis [268].

For example, Huang et al. [121] demonstrated that dynamic compression applied to rabbit BMSCs embedded in a 2% agarose construct, in the absence of exogenous TGF-β, significantly enhanced the gene expression of collagen II, aggrecan, and TGF-β1 genes in the loaded groups compared to the free-swelling control without TGF-β. This finding indicates that dynamic compression alone (i.e., in the absence of exogenous growth factors) can promote a chondrogenic gene expression profile [121]. Furthermore, Huang et al. [122] demonstrated that dynamic compression, also in the absence of TGF-β supplementation, induced a pronounced upregulation of chondrogenic genes using the same TE construct, particularly with 2 or 4 h of loading. In the case of 4 h of loading, effects were only detected after the second day. The study also revealed distinct temporal expression patterns of both chondrogenic and TGF-β signalling genes, along with their corresponding proteins [122]. Mauck et al. also demonstrated that, after 14 and 28 days, dynamic compression in the absence of TGF-β supplementation significantly increased sGAG content compared to the free-swelling control in bovine BMSCs embedded in a 2% agarose [201]. Terraciano et al. also demonstrated that dynamic compression significantly enhances Sox9 and aggrecan gene expression compared to free-swelling controls, in the absence of TGF-β supplementation in goat BMSC-encapsulated in poly(ethylene glycol)-diacrylate hydrogels (PEGDA) [290]. Moreover, Kisiday et al. showed that dynamic compression, also without TGF-β supplementation, significantly enhanced GAG accumulation in equine BMSCs cultured in 2% agarose compared to the free-swelling control [149]. Furthermore, Huang et al. found that dynamic compression without TGF-β supplementation (and also without significant TGFβ-free preculture time) substantially reduced GAG synthesis compared to the free-swelling control in bovine BMSC seeded in 4% agarose hydrogels [120]. In Schätti et al.’s study, dynamic compression alone had no effect on hBMSC chondrogenesis in fibrin–PU scaffolds relative to free-swelling group [268]. Moreover, Mouw et al. implemented a 16-day TGF-β-free preculture period and observed a substantial increase in pSMAD2/3 protein levels [216].

- AChs

Studies involving AChs demonstrated that dynamic compression enhances chondrogenic performance, as evidenced by increased expression of chondrogenic genes [224, 225] and greater GAG synthesis [41, 202, 297, 310] compared to the free-swelling control. However, a study by Hunter et al. reported a contrasting outcome when compared to Chai et al. and Mauck et al. (bovine AChs-seeded in 2% Agarose) as well as Waldman et al. (bovine AChs-seeded on Calcium phosphate substrate), where dynamic compression significantly reduced GAG production compared to the free-swelling control in bovine AChs-seeded in fibrin gel [125].

When comparing dynamic and static compression, the literature shows that dynamic compression promotes higher GAG synthesis [60, 125, 161] and chondrogenic gene expression profile [333] compared to static compression.

For instance, Lee et al. also demonstrated that dynamic compression, specifically at a frequency of 1 Hz, significantly enhanced sGAG synthesis, whereas static compression substantially reduced it compared to the free-swelling control in bovine AChs-seeded in 3% Agarose. Interestingly, this enhancement was observed only at 1 Hz; lower (0.3 Hz) and higher (3 Hz) frequencies either resulted in a substantial reduction or no significant effect on sGAG synthesis relative to the free swelling group[161]. Davisson et al. found that dynamic compression, especially at 50% strain, significantly promoted sGAG synthesis when compared to static compression and free-swelling control. Additionally, static compression was reported to reduce sGAG synthesis relative to free swelling in chondrocytes cultured on a PGA scaffold following a preculture period without TGF-β supplementation in bovine AChs-seeded in PGA scaffold [60]. Hunter et al. also observed that the 0.1 Hz dynamic compression group had significantly higher overall GAG content compared to the static compression group; however, both compression conditions showed a pronounced decrease in GAG content when compared to the free-swelling control in bovine AChs-seeded in fibrin gel [125]. More recently, Xu et al. reported that dynamic compression significantly upregulated COL2A1, ACAN, PRG4, and SOX9 compared to both static compression and the free-swelling control. Notably, static compression also induced a marked increase in chondrogenic gene expression relative to free swelling in ATDC5- encapsulated in GelMA hydrogels [333].

Studies investigating the effects of intermittent versus continuous loading have reported inconsistent findings, highlighting the need for further research. In favour of intermittent loading, Chowdhury et al. reported that bovine AChs seeded in 3% agarose exhibited enhanced sGAG synthesis compared to continuous loading [51, 52]. Nicodemus and Bryant [231], working with bovine AChs-seeded in PEG hydrogels, found that intermittent compression led to upregulation of collagen II and MMP gene expression relative to continuous loading,however, total GAG content was significantly higher under continuous loading [231]. Xie, Jun et al. demonstrated that 24-h continuous loading significantly increased chondrogenic and fibrocartilaginous gene expression levels compared to intermittent compression in rabbit AChs seeded onto poly(L-lactide-co-epsilon-caprolactone) scaffold [330]. In line with this study, even though both studies used different ACh sources and different biomaterial, Ng et al. also demonstrated that continuous loading for 3 or 6 h resulted in greater collagen type II and IX deposition compared to both intermittent loading (1 h on/1 h off) and the free-swelling control in bovine AChs-seeded in 2% Agarose [229].

## Exogenous TGFβ-supplemented culture media

- BMSCs

In the presence of TGF-β supplementation, and without a significant TGF-β-supplemented preculture period, several studies [11, 110, 121, 290, 292] have demonstrated that dynamic continuous compression further induces chondrogenic differentiation. However, four studies reported a significant reduction in GAG and collagen content [120, 149, 293, 294], as well as in the expression of chondrogenic genes [87], compared to the free-swelling control.

In studies showing positive outcomes associated with dynamic compression, such as the case of, Angele et al. reported a pronounced upregulation of collagen II and aggrecan gene expression on day 7 in human BMSCs embedded in a hyaluronan–gelatin composite. By day 21, consistent with these earlier findings, a substantial increase in both proteoglycan and collagen content was observed [11]. Moreover, Huang et al. showed a pronounced increase in collagen II gene expression compared to the free-swelling control supplemented with TGF-β in rabbit BMSC-seeded in 2% Agarose [121]. Additionally, Terraciano et al. demonstrated that 2.5 h of dynamic compression significantly upregulated Sox9, aggrecan, and collagen II gene expression and increased GAG production compared to the free-swelling control with TGF-β supplementation in goat BMSC-seeded in PEGDA hydrogel [290]. Furthermore, Haugh et al. demonstrated that dynamic compression significantly increased collagen II and aggrecan gene expression in both core and annular region of hBMSC-laden 2% agarose constructs. Interestingly, the annular region showed a substantial downregulation of COL1A1 expression [110]. Ultimately, Thorpe et al. confirmed earlier findings that dynamic compression significantly upregulates COL2A1 while downregulating COL1A1. However, their results for GAG and collagen content were contradictory, showing a reduction in these matrix components in porcine BMSC-seeded in fibrin gel [292].

Exploring determinant factors such as the presence of a TGF-β-supplemented preculture period, the majority of studies have demonstrated that this preculture enhances both chondrogenic gene expression and ECM-specific matrix production compared to the free-swelling control [216, 266, 345, 346] or immediate dynamic compression without preculture [87]. However, two studies reported no significant effect of dynamic compression following this preculture period compared to the free-swelling control [120, 294]. Notably, Thorpe et al. showed that including a TGF-β-supplemented preculture period along with dynamic compression significantly increased GAG and collagen content compared to omitting this preculture in the dynamic compression group [294].

Among the studies demonstrating the benefits of a TGF-β-supplemented preculture period, Mouw et al. reported that dynamic compression, when applied following a TGF-β-supplemented preculture period, significantly increased collagen II and aggrecan gene expression, as well as sGAG and total protein synthesis, compared to the free-swelling control, in bovine BMSCs embedded in 3% agarose[216]. Moreover, Zhang et al. reported that dynamic compression significantly enhanced collagen II and aggrecan gene expression, as well as markedly increased sGAG and type II collagen (COLII) content, compared to the free-swelling control in hBMSCs seeded on poly L-lactide-co-caprolactone (PLCL)/chitosan scaffolds. In addition, collagen X and RUNX2 expression were substantially decreased relative to the free-swelling control, suggesting that, in this experimental setup, dynamic compression contributes to the suppression of hypertrophic differentiation [345, 346].Moreover, Sawatjui et al. reported that dynamic compression enhanced aggrecan and GAG accumulation compared to the free-swelling control in hBMSC seeded on Silk-fibroin or Silk fibroin-gelatin/chondroitin sulfate/hyaluronate scaffolds [266]. Ge et al. found that applying dynamic compression immediately reduced the expression of chondrogenic genes in hSMSC-seeded in 2% agarose. In contrast, when compression was delayed, it significantly increased SOX9 activity and chondrogenic gene expression, while reducing the expression of genes related to hypertrophy, fibrocartilage, osteogenesis, and matrix degradation [87].

The subcellular mechanobiological mechanisms behind these responses are still poorly explored, and studying them could clarify the conflicting results, beyond the influence of known confounding factors. It is hypothesized that the presence of a TGF-β-supplemented preculture period promotes the formation of the PCM, which may alter how mechanosensitive receptors perceive dynamic compression, ultimately triggering distinct mechanotransduction pathway, even if the resulting chondrogenic outcomes appear similar.

- AChs

In studies involving AChs with a preculture period including TGF-β, about half demonstrated improved chondrogenic potential, whereas the remaining studies reported no significant differences compared to free-swelling control.

In those studies showing positive chondrogenic responses, Sawatjui et al. demonstrated that dynamic compression increased collagen type II, aggrecan and GAG accumulation compared to the free-swelling control in hAChs seeded on Silk-fibroin or Silk fibroin-gelatin/chondroitin sulfate/hyaluronate scaffolds [266]. Additionally, Xie et al. reported that dynamic compression enhanced the expression of COL2, ACAN, and SOX9 compared to the free-swelling control in hAChs encapsulated in PEG-PDLLA/PEG-PCL hydrogel [331]. Lima et al. (bovine AChs) and Bian et al., (canine AChs) using 2% agarose-based scaffolds, reported no significant differences in GAG or collagen content when compared to the free-swelling control [20, 184].

### b. Shear stress

Shear stress is applied along the horizontal plane, causing lateral displacement of the upper surface in the opposite direction of the bottom surface. There are two types of shear stress: fluid flow-induced shear stress (i.e., fluid shear) and contact shear, which occurs when a solid object slides along the surface of the TE construct. There are various types of shear-induce systems, such as perfusion bioreactors, spinner flask and contact shear bioreactors. ACh studies investigating fluid shear have shown that fluid flow enhances sGAG and Collagen synthesis relative to the free swelling condition in bovine AChs using PGA scaffold [91, 245]. Additionally, Raimondi et al. demonstrated that at a flow rate of 0.5 mL/min, the sGAG/DNA ratio in hACh[256] and the collagen type II to collagen type I ratio in bovine ACh using DegraPol foam [255] were higher under low shear stress conditions (4.6 or 1.2 mPa, respectively) compared to both higher shear stress levels (14, 25, and 56 mPa, or 6.6 mPa, respectively) and the free-swelling control, suggesting that lower shear stress may promote a more favourable cartilaginous matrix production. Moreover, Yu et al. reported that fluid shear induced by a flow rate of 3mL/min significantly enhanced the expression of chondrogenic markers, including collagen II and aggrecan, compared to the free-swelling control in bovine ACh-seeded in 2% alginate [339]. Mizuno et al. observed that fluid shear induced by a flow rate of 0.33 mL/min significantly reduced sGAG content and substantially decreased aggrecan and collagen II gene expression compared to the free-swelling control in bovine AChs-seeded in collagen sponge [213]. With respect to contact shear, studies have reported increased proteoglycan content compared to the free-swelling control [308, 308]. Additionally, higher sliding velocity (i.e., 28 mm/s, compared to 0.28 and 2.8 mm/s) was associated with a significant upregulation of lubrication-related gene expression and increased release of their corresponding proteins, including cartilage oligomeric matrix protein (COMP), proteoglycan 4 (PRG4), and hyaluronan synthase 1 (HAS1) in bovine AChs-seeded in fibrin PU scaffolds [321].

### c. Combination of compression and shear

Although most mechanobiology studies focus on the effects of a single biophysical stimulus, some have investigated whether combining multiple forces may enhance chondrogenic outcomes or prove more effective than applying a single stimulus alone.

- MSCs

In studies using hBMSC, Li et al. were among the first to demonstrate that the combination of dynamic compression and contact shear, even in the presence of low concentrations of TGF-β1 or without its supplementation, significantly upregulated chondrogenic and hypertrophic gene expression and markedly increased sGAG synthesis compared to the free-swelling control in fibrin-PU scaffolds [172, 179, 180]. A subsequent study by Li et al. demonstrated that higher compression frequency and strain led to a pronounced increase in sGAG synthesis, along with upregulated expression of chondrogenic, fibrocartilaginous, and TGF-β–related genes compared to the free-swelling control [172, 179, 180]. These findings influenced subsequent studies, all of which omitted TGF-β1 supplementation from the culture media. Further research investigated whether the combination of compression and shear would result in superior chondrogenesis compared to the application of either force alone. Although sGAG synthesis did not show significant differences between the combined and individual force groups, gene expression analysis of chondrogenic markers revealed that the combined application of compression and shear induced significantly greater upregulation compared to either stimulus alone or the free-swelling control. These findings suggest that a synergistic mechanical stimulation protocol may more effectively promote chondrogenesis [268]. Three additional studies further supported that the combination of dynamic compression and contact shear promotes chondrogenesis of hBMSCs seeded in fibrin–polyurethane scaffolds[82, 83, 158] or methylcellulose–polyurethane constructs[56].

- AChs

Waldma et al. and Shahin et al. showed that combined dynamic compression and contact shear showed to significantly enhance proteoglycan and collagen content [274, 307]. Furthermore, in two separate studies, Grad et al. demonstrated that the combination of dynamic compression and shear induces a more pronounced chondrogenic phenotype compared to dynamic compression alone. This was evidenced by significantly higher expression levels of PRG4, COMP, aggrecan and COL2, increased release of PRG4, HA and COMP proteins, and the presence of lubricin exclusively in the synergistic loading condition in hAChs/bAChs-seeded in fibrin PU scaffolds [94, 95]. Meinert et al. also observed an increase in COL2A1 expression and COLII deposition, along with a decrease in COL10A1, when 30% dynamic compression superimposed with sliding shear was applied to hACs embedded in a GelMA-HAMA hydrogel, following a preculture period with TGF-β [207]. Another study by Paggi et al., using their own developed cartilage-on-chip device, showed that synergistic mechanical stimulation (compression and shear) increased COL2A1gene expression, GAG synthesis, as well as Aggrecan, COL II, and COL IV deposition compared to the free-swelling control in hAChs encapsulated in an agarose-based scaffold [236].Three studies reported that combined mechanical stimulation protocols (dynamic compression combined with either contact shear or fluid shear) did not result in statistically significant differences in GAG or collagen content, nor in chondrogenic gene expression, compared to the free-swelling control [20, 97, 249]. Additionally, a study by Seidel et al. demonstrated that the combined mechanical stimulation protocol (compression and shear) significantly reduced collagen content compared to the free-swelling control [271].

Here, we present the current insights most strongly supported by published studies and highlight areas with conflicting findings:

- Without exogenous TGFβ supplementation or an extended TGFβ-free preculture, dynamic compression has unclear effects on BMSC chondrogenesis [120–122, 149, 201, 268] and ACh chondrogenic performance [41, 60, 125, 125, 161, 202, 224, 225, 297, 310]. Conflicting findings leave it uncertain whether intermittent or continuous loading better supports AChs [51, 52, 229, 231, 330]. Further research with controlled confounding variables is needed to clarify the effects of dynamic compression and its specific protocols on MSC and chondrocyte chondrogenesis.
- When exogenous TGF-β is present during both preculture and loading, the impact of dynamic compression on BMSC chondrogenesis [11, 87, 110, 121, 149, 290, 292–294] and ACh chondrogenic potential [20, 184, 266, 331] remain uncertain due to inconsistent findings, highlighting the need for further research.
- Conflicting results on the ACh chondrogenic role of fluid flow shear indicate that more research is needed [91, 213, 245, 255, 308, 308, 309, 309].
- Dynamic compression combined with shear promotes hBMSC chondrogenesis without exogenous TGFβ supplementation [56, 82, 83, 158, 172, 179, 180, 268]. For AChs, the influence of combined dynamic compression and shear on chondrogenic potential remains uncertain, as studies report conflicting results, highlighting the need for further research [20, 94, 95, 97, 207, 236, 249, 271, 274, 307].

### Tackling post-traumatic osteoarthritis

**Fig. 5:**
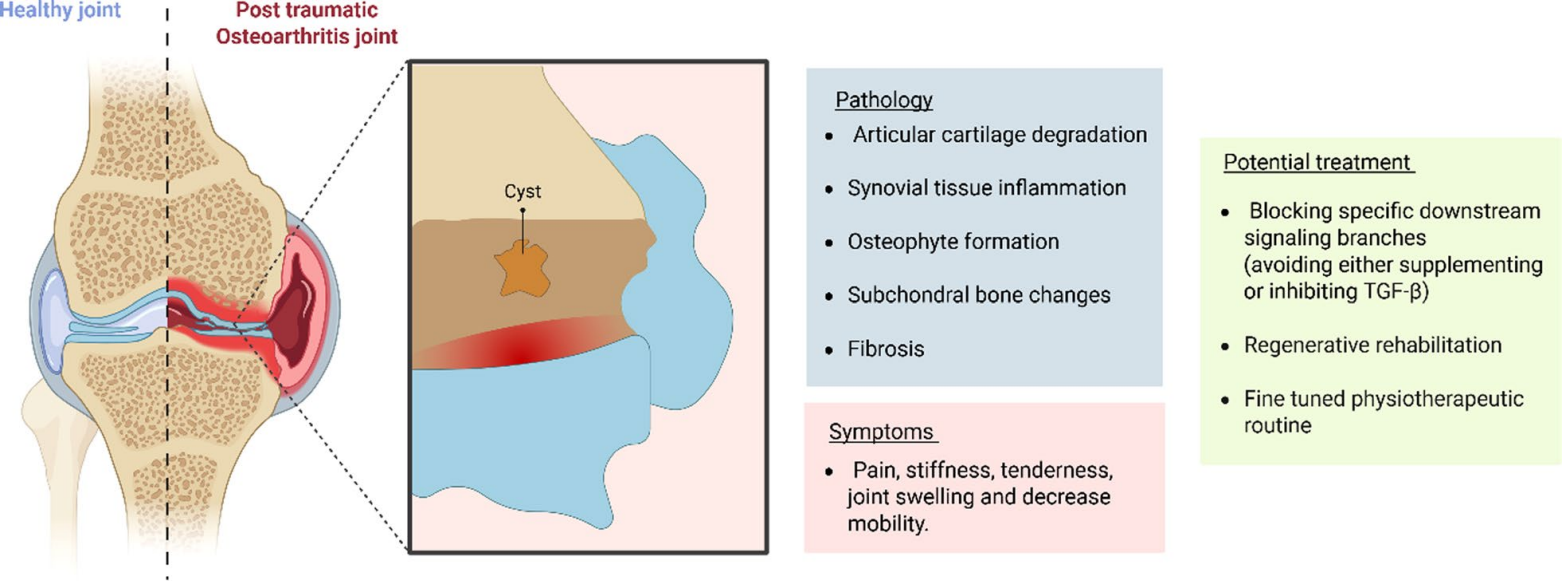
Post-traumatic osteoarthritis pathologies, symptoms and future treatments

Post-traumatic osteoarthritis (PTOA) is a subtype of osteoarthritis (OA), accounting for about 12% of OA cases, and is characterized by being triggered by a traumatic injury to the joint [33]. Similar to other forms of OA, PTOA (Fig. 5) is a heterogeneous disease characterized by AC degradation, synovial tissue inflammation, osteophyte formation, subchondral bone changes (i.e., sclerosis, cyst formation and accelerated remodelling), and fibrosis, ultimately leading to reduced joint mobility and function [155]. Regarding synovial tissue inflammation, interleukin-1β (IL-1β) and tumour necrosis factor-α (TNF-α) are considered key pro-inflammatory cytokines that stimulate chondrocytes to produce nitric oxide (NO), activate MMPs leading to cartilage degradation, and suppress the synthesis of ECM proteins [26]. Clinical symptoms of PTOA commonly include pain, stiffness, tenderness, joint swelling, and decreased mobility. While PTOA primarily affects young adults following traumatic, often sports-related injuries, primary OA is generally associated with older age; however, aging has also been shown to increase the risk of PTOA progression [47]. This highlights the importance of understanding the cellular mechanisms underlying PTOA initiation and progression, not only to enable early detection but also to optimize therapeutic strategies for its prevention and treatment. Although, as previously mentioned, (non-traumatic) OA and PTOA share similar macroscopic pathological features, studies have shown that they may differ at the molecular level [186]. Currently, there are no effective disease-modified therapies for the cure of PTOA or OA; therefore, treatment is focused on symptom management through pharmacological agents, physical therapy, and lifestyle adjustments [144]. In cases where these approaches are insufficient, joint replacement surgery may be undertaken. This highlights the urgent need for the development of novel therapeutics. Due to the limited number of studies specifically addressing each OA subtype—and the few that focus exclusively on PTOA—the following paragraphs, which highlight current findings on underlying cellular mechanisms and potential therapies, will focus on OA in general.

#### a. OA Pathological subcellular components and mechanisms

Starting with the primary cellular mechanosensory components, studies have demonstrated that OA chondrocytes exhibit an altered integrin profile, characterized by elevated levels of α1β1, α3β1, α2β1, α4β1, and α6β1 compared to normal chondrocytes; however, the functional significance of these changes is not yet fully understood [190]. Additionally, gene expression levels of αvβ3 integrins have been shown to be increased in the synovial membranes of OA joints. Wang et al. also demonstrated that αvβ3-mediated signalling, which occurs in response to chondrocyte breakdown, activates the ERK pathway and subsequently induces the expression of matrix MMPs in synovial membrane and fluid in OA, thereby contributing to OA progression. The same study demonstrated that inhibition of FAK, a downstream effector of integrin signalling, attenuated OA progression. Overall, these findings suggest that both αvβ3 integrins and FAK play critical roles in the pathogenesis of OA [315]. Additionally, deletion of the focal adhesion protein Kindlin-2 in AChs has been shown to lead to spontaneous OA and to exacerbate lesions in induced OA mouse models [326, 327], suggesting that Kindlin-2 has a key role in OA development and progression. When considering the actin cytoskeleton and its actin-binding proteins, particularly Adseverin, one study showed that deletion of Adseverin in a mouse model led to increased cartilage stiffness, thinner hyaline cartilage, thicker calcified cartilage zones, and enhanced hypertrophic differentiation. It also reported increased OA severity in a medial meniscus (DMM)-induced OA mouse model, suggesting that Adseverin not only modulates cartilage homeostasis but also regulates OA progression [43].

Among mechanosignalling pathways, TGF-β signalling is involved in OA development and progression. Mutations in SMAD3 were found in patients with early- onset OA [302] and polymorphism in SMAD3 have been associated with knee and hip OA [301]. Moreover, deficiency of Smad3 [336], Tgfβr2 [277] or Alk5[316] in AChs in mice resulted in an OA -like phenotype. Additionally, under physiological conditions, levels of active TGFβ in the joint are very low. Albro et al. have shown that the concentration of active TGFβ in the human synovial joint is approximately 0.03 ng/mL [4]. However, Fava et al. reported that this level of active TGFβ is significantly elevated in the synovial fluid of OA patients, reaching up to 3.8 ng/mL [72]. Although macrophages and fibroblasts can also produce TGF-β, [163, 206, 210], cartilage breakdown–associated enzymatic activity likely drives the release and activation of ECM-bound TGF-β. [355]. Therefore, regulating the concentration of active TGF-β may represent a potential therapeutic strategy. In support of this, as mentioned in a previous section, Wang et al. demonstrated using bovine AChs-seeded in 2% agarose hydrogels that lower doses of active TGF-β (0.1–1 ng/mL) reduce the deposition of type I collagen, mitigate the formation of cell clusters—a phenomenon characteristic of OA pathology [191]— without suppressing collagen synthesis as observed at supraphysiological concentrations [317]. Although this study did not investigate the molecular mechanisms underlying these dose-dependent phenotypic changes in AChs, earlier work in human fibroblasts may provide some insight. Remst et al. found that high concentrations of active TGF-β (above 5 ng/mL) preferentially stimulate phosphorylation of SMAD1/5/8, whereas lower concentrations predominantly activate the SMAD2/3 signalling pathway [257]. Extrapolating from these findings, and in light of the observations by Weng et al., it appears that in healthy joints—where active TGF-β levels are low—the SMAD2/3 pathway (via ALK5) is preferentially activated. In contrast, in OA joints with elevated TGF-β concentrations, activation shifts toward the SMAD1/5/8 pathway (via ALK1). Furthermore, in line with this rationale, a study showed that ALK1/ALK5 ratio increases with OA progression [25]. The elevated concentrations of TGF-β observed in the joints of OA patients are likely to contribute to, or even trigger, pathological features such as synovial fibrosis, osteophyte formation, subchondral bone alterations, and a hypertrophic chondrocyte phenotype. Supporting this, studies using TGF-β inhibitors have demonstrated a mitigation of these OA-related pathologies [27, 267, 351]. This suggests that blocking TGF-β activity could represent a potential therapeutic strategy for OA. However, while both systemic and joint-localized TGF-β inhibition appear capable of attenuating OA pathology, they may also negatively affect healthy cartilage. In addition, since TGF-β plays a key role in suppressing inflammation, its inhibition could exacerbate inflammatory responses, as observed in studies using TGF-β1-deficient mice[165]. Other approaches, based on a different rationale, aim to stimulate the TGF-β pathway to promote cartilage repair [286, 345, 346]. However, as mentioned above, elevated TGF-β activity can exacerbate other osteoarthritic pathologies, aside from cartilage degradation. Therefore, instead of inhibiting or activating the TGF-β pathway, a more selective strategy—such as blocking specific downstream signalling branches like the SMAD1/5/8 pathway—may represent a safer and more effective therapeutic approach. Combining both integrin and TGF-β signalling factors, Zhen et al. demonstrated that deletion of αv in an OA mouse model prevents excessive TGF-β activation and thereby attenuates cartilage degradation. This strategy is based on the rationale discussed earlier, as αv is known to mediate the activation of latent TGF-β. Nevertheless, it shares the same limitation mentioned above, as it may also reduce TGF-β’s beneficial effects on cartilage homeostasis [350].

Besides the TGF-β-induced SMAD pathway, studies have suggested that SMAD-independent pathways (such as those highlighted in this review) may also be involved in the onset and progression of OA and could serve as promising targets for new therapeutic approaches [32, 85, 285]. In addition, multiple studies have shown that the YAP/TAZ pathway also appears to play a role in OA progression; however, the results remain conflicting regarding whether YAP exerts a pro- or anti-inflammatory effect in OA. Zhang et al. reported a pronounced increase in YAP nuclear localization in human OA cartilage compared to healthy cartilage, where YAP was predominantly localized in the cytoplasm. Supporting this observation, they also examined CTGF protein levels (a well-known YAP target) and found increased expression in human OA cartilage. Furthermore, they demonstrated that pharmacological inhibition of YAP using Verteporfin enhanced COL II deposition, attenuated cartilage degeneration, and promoted YAP cytoplasmic localization in human OA cartilage, suggesting that inhibition of YAP activity may alleviate OA pathology [347]. Gong et al. additionally demonstrated that YAP expression is significantly upregulated in human OA chondrocytes compared to healthy chondrocytes [90]. Conversely, Deng et al. observed a reduction in YAP expression levels in human OA cartilage, which decreased progressively with increasing disease severity and higher OARSI grades. In the same study, they reported that YAP activation attenuated cartilage degradation during OA progression in a murine model [64]. Given these conflicting findings, further research is necessary to establish a clearer understanding of YAP's role in OA pathogenesis and to explore its potential as a therapeutic target.

#### b. Pathological loading and Mechanotherapy

Despite significant efforts over the past decades to identify key molecular pathways involved in OA and to develop corresponding biochemical therapies, the potential of mechanical stimulation (either as a standalone treatment or in combination with molecular approaches) has often been overlooked in the development of therapeutic strategies. However, emerging evidence suggests that physiological mechanical stimulation induces anti-inflammatory and anabolic responses in either AChs-based TE constructs or AC under inflammatory conditions [51, 52, 295], and enhances anabolic activity in OA AChs-based TE constructs [133, 134, 203].

For instances, Chowdhury et al. observed that dynamic compression inhibits IL-1β induced release of NO and prostaglandin E_2_(PGE_2_) in bovine ACh-seeded in 3% agarose hydrogels [51, 52]. In addition, Torzilli et al. show that uniaxial compression load performed to bovine AC suppressed IL-1β induced matrix degradation [295]. Furthermore, studies conducted using OA chondrocytes seeded in alginate gels reported that dynamic compression enhances the chondrogenic phenotype, as shown by a significant upregulation of ACAN, COL2A1, COL1A1, COL10A1, and PRG4 only 2 h after a single 1 h loading, especially in the superficial zone-derived chondrocytes. In addition, ACAN and PRG4 gene expression levels were shown to be dependent on both strain magnitude and load duration, with 50% dynamic compression strain and a 3-h loading cycle yielding the highest expression levels [133]. Notably, introducing a preculture period prior to loading further enhanced the expression of chondrogenic genes [134]. Fluid shear in the study by Mayer et al. was shown to promote collagen type II synthesis and suppress collagen type I in OA chondrocytes seeded into collagen sponges [203].

While physiological loading can improve OA-related pathologies, excessive or abnormal mechanical loading has catabolic effects, increasing the production and activity of proteolytic enzymes and leading to ECM degradation [67, 156, 196, 342]. In light of these findings, it is crucial to conduct further research to identify the optimal physiological mechanical loading parameters that most effectively support the restoration of cartilage integrity in OA patients. This approach, commonly known as rehabilitation therapy or mechanotherapy, has shown encouraging results in clinical studies, primarily based on questionnaires reporting improvements in physical function and quality of life [70, 217]. However, structural outcomes, such as those assessed by MRI or other medical examinations, remain insufficiently explored [129]. In contrast, preclinical studies using OA murine models have investigated these structural effects more extensively. For instance, Zheng et al. demonstrated that dynamic loading protects against cartilage degradation, enhance chondrocyte proliferation, decrease MMP13 expression, prevent osteophyte formation, reduce synovial inflammation, and regulate abnormal bone remodelling in an OA mouse model [352].

Here, we focus on the cellular components and mechanisms linked to OA, as well as insights into potential mechanotherapy strategies:

- αVβ3 integrin, FAK, Kindlin-2, and Adseverin play essential roles in the development and progression of OA [43, 315, 326, 327].
- Active TGF-β levels are elevated in OA synovial fluid, likely due to enzymatic release of ECM-bound latent TGF-β during cartilage breakdown.
- TGF-β pathway is linked to OA, emphasizing the critical role of this pathway in OA pathology [277, 301, 302, 316, 336]. While TGF-β signaling is involved in OA, systemic inhibition risks damaging healthy cartilage, making selective targeting of SMAD1/5/8 a promising therapeutic approach.
- YAP’s involvement in OA is not fully understood, highlighting the need for further research to assess its therapeutic potential [64, 90, 347].
- Physiological mechanical stimulation promotes anti-inflammatory and anabolic responses under OA-like inflammatory conditions [51, 52, 133, 134, 203, 295].
- More research is needed to evaluate the structural outcomes of mechanotherapy in clinical settings.

## Conclusions and Perspectives

Mechanobiology has rapidly emerged as a growing research field, especially over the last decade, aiming to understand how mechanical cues—such as matrix-related signals and external force stimuli—are perceived and integrated by chondrogenic cells to regulate their biological functions. This review aimed to address a key gap in chondrogenic mechanotransduction and mechanically induced chondrogenic mechanobiology. While most studies have focused on cell–matrix interactions through biomaterial design—such as adjusting stiffness, cell binding, topography, porosity or viscoelasticity—the role of external mechanical forces has received much less attention. To provide insight into the current understanding of chondrogenic cell mechanosensing, this review highlights key mechanically responsive structures involved in mediating external forces, such as β1 integrins and FAK, which have been shown to mediate chondrogenic responses to dynamic compression. Furthermore, associated signalling molecules, including talin and vinculin, have been identified as central mediators of the cellular response to mechanical stimulation. This review highlights both the influence of mechanical stimulation on cytoskeletal organization and the association between cytoskeletal organization and chondrogenic outcomes. Mechanical loading, such as dynamic compression, has been shown to promote cortical actin arrangement and F-actin depolymerization in hBMSCs. Additionally, cortical-like actin arrangements are linked to a stable chondrogenic phenotype, while prominent stress fibres promote hypertrophic differentiation. Herein, we also highlighted that the principal mechanism of ECM-bound LTGF-β activation is mechanical loading, whereas integrins and proteases play a lesser role. Mechanistically, LTGF-β activation is mediated through talin-dependent cytoskeletal reorganization, which generates cytoskeletal tension, promoting the binding of αv integrins to LAP and ultimately leading to LTGF-β activation. Moreover, LTGF-β activation triggers phosphorylation of downstream pathways such as SMAD, Rho-GTPase, MAPKs, PI3K/AKT/mTOR, MRTF-SRF, and YAP/TAZ, thereby modulating chondrogenic cell behaviour. SMAD2/3 and SMAD1/5/9 signalling via ALK5 and ALK1are necessary for hBMSC chondrogenesis. Notably, p38 and PI3K act as positive regulators of MSC chondrogenesis, whereas ERK2 functions as a negative regulator. In the case of YAP, it promotes chondrocyte proliferation and early chondrogenesis. Furthermore, mechanical stimulation appears to mediate RhoA activity and YAP translocation. Additionally, this review explores in vitro studies investigating an alternative approach to cartilage repair that integrates recent advances in chondrogenic mechanobiology by combining tissue engineering constructs with rehabilitation protocols, known as Regenerative Rehabilitation (RR). Herein, we highlight the potential of tissue engineering (i.e., stiff hydrogel for chondrocytic cells and faster relaxing, viscous hydrogel for MSCs) and summarize various mechanical loading regimens (i.e., dynamic compression combined with shear stress enhances hBMSC chondrogenic outcome) applied in vitro using these constructs as models. This knowledge, while emphasizing the need to optimize mechanical parameters for specific tissue-engineered constructs to achieve neocartilage repair with a hyaline-like phenotype, also advances the field, paving the way for studies in large animal models and clinical trials. Lastly, the review addresses the mechanobiology of OA (e.g., emphasizing the essential roles of αVβ3 integrins, FAK Kindlin-2, and Adseverin in disease development and progression), and evaluates in vitro rehabilitation-based strategies, such as dynamic compression, which enhance the chondrogenic phenotype of OA chondrocytes. Despite notable advancements in chondrogenic and OA-related mechanobiology, further research remains necessary. Cutting-edge technologies, such as single-cell RNA sequencing, spatial omics, and the continued development of bioreactor systems, now make it possible to investigate more localized outcomes within tissue-engineered constructs, as well as the complex cellular crosstalk within both healthy and diseased AC [200]. A major challenge in the field, which could significantly influence future developments in regenerative rehabilitation, is the absence of standardized protocols and guidelines for studies combining tissue engineering and mechanical stimulation for cartilage regeneration. Implementing such standards would help clarify whether observed effects arise from mechanical loading or the biomaterial itself by minimizing confounding factors such as cell source and species, media composition, and the presence or absence of chondrogenic factors. Future research should focus not only on enhancing our understanding of chondrogenic mechanotransduction and creating more physiologically relevant in vitro cartilage models but also on developing RR therapies with strong potential for clinical application.

## Declarations

### Availability of data and materials

No datasets were generated or analysed during the current study.
